# Practical Synthesis of Polyamine Succinamides and Branched Polyamines

**DOI:** 10.1002/open.202200147

**Published:** 2022-10-25

**Authors:** Abdulaziz H. Alkhzem, Maisem Laabei, Timothy J. Woodman, Ian S. Blagbrough

**Affiliations:** ^1^ Department of Pharmacy and Pharmacology University of Bath Claverton Down Bath BA2 7AY UK; ^2^ Department of Biology and Biochemistry University of Bath Claverton Down Bath BA2 7AY UK

**Keywords:** antibiotics, polyamines, spermine, succinic anhydride, thermine

## Abstract

Antibiotic resistance is now a growing threat to human health, further exacerbated by the lack of new antibiotics. We describe the practical synthesis of a series of substituted polyamine succinamides and branched polyamines that are potential new antibiotics against both Gram‐positive and Gram‐negative bacteria, including MRSA and *Pseudomonas aeruginosa*. They are prepared via 1,4‐Michael addition of acrylonitrile and then hydrogenation of the nitrile functional groups to primary amines. They are built upon the framework of the naturally occurring polyamines thermine (3.3.3, norspermine) and spermine (3.4.3), homo‐ and heterodimeric polyamine succinic amides. Linking two of the same or different polyamines together via amide bonds can be achieved by introducing a carboxylic acid group on the first polyamine, then coupling that released carboxylic acid to a free primary amine in the second polyamine. If the addition of positive charges on the amino groups along the polyamine chains are a key factor in their antimicrobial activity against Gram‐negative bacteria, then increasing them will increase the antimicrobial activity. Synthesising polyamine amide dimers will increase the total net positive charge compared to their monomers. The design and practical synthesis of such homo‐ and hetero‐dimers of linear polyamines, spermine and norspermine, are reported. Several of these compounds do not display significant antibacterial activity against Gram‐positive or Gram‐negative bacteria, including MRSA and *Pseudomonas aeruginosa*. However, the most charged analogue, a branched polyamine carrying eight positive charges at physiological pH, displays antibiofilm activity with a 50 % reduction in PAO1 at 16–32 μg mL^−1^.

## Graphical abstract

**Building upon the framework** of naturally occurring polyamines, the practical synthesis of a series of substituted homo‐ and hetero‐dimers of linear polyamines, spermine and norspermine succinamides and branched polyamines is achieved via 1,4‐Michael addition of acrylonitrile and then hydrogenation of the nitrile functional groups to primary amines. The most charged analogue, a branched polyamine carrying eight positive charges at physiological pH, displays antibiofilm activity with a 50 % reduction in PAO1 at 16–32 μg mL^−1^.

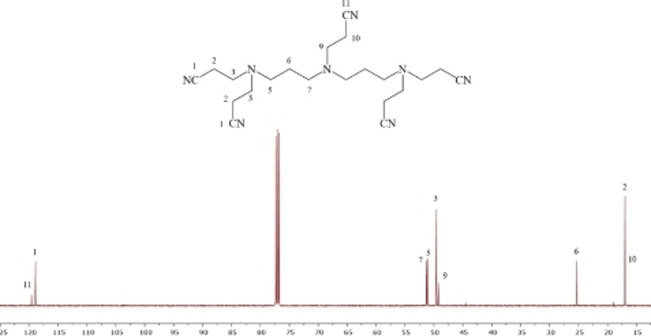

## Introduction

*Staphylococcus aureus* and *Pseudomonas aeruginosa* are recognized to be the leading causes of infections in community and health facilities. Such an infection is particularly lethal in hospital environments where Meticillin‐resistant *S. aureus* (MRSA) claims 64 % higher mortality than observed in patients that get infected with the Methicillin‐sensitive *S. aureus* (MSSA). The threat of antimicrobial resistance is therefore ever growing, and strategies are needed to develop new molecules with novel modes of action to curtail infections. The action of polyamines on biofilms differs in different bacterial species and strains. For example, for *Vibrio cholerae*, Karatan and co‐workers have established how spermidine (3.4) and spermine (3.4.3) regulate and disrupt biofilm formation via transport and signaling pathways.[^1^, ^2^] Likewise, Woster and colleagues have addressed this important problem with polyamine conjugates derivatised into antibacterial diamines which target bacterial membranes.^[3]^ Haldar and co‐workers have independently reported positively charged membrane active (di)Phe and (di)Lys conjugated lipophilic norspermidine (3.3) amides with selective antibacterial activity.[^4^, ^5^] The p*K*_a_ values for polyamine norspermine (3.3.3, thermine) are 10.6, 10.5, 8.7, and 6.7 (potentiometry) at physiological pH 7.4.[^6^, ^7^] If the addition of further positive charges on the amino groups along the polyamine chains are a key factor in their antimicrobial activity against Gram‐negative bacteria, then increasing them may well increase their antimicrobial activity.

**Figure 1:**
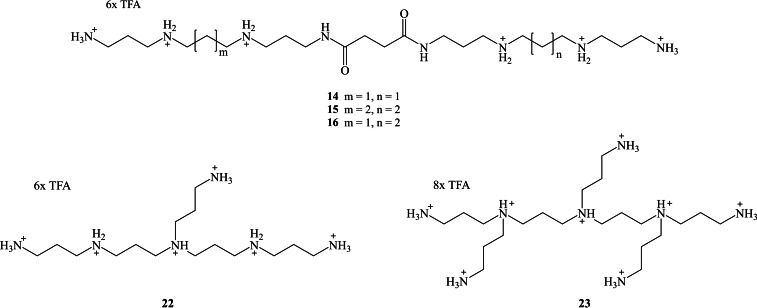
Target compounds, linear **14**, **15**, **16**,and branched polyamines **22** and **23**.

The synthesis of compounds containing two pharmacophores linked by a simple or longer linker has become a promising approach to not only minimize the drawbacks of the medications, but also to improve their affinity and potency. This hybrid drug approach was triggered by numerous attempts at discovering novel artificial scaffolds that could produce antibiotics with the ability to overcome drug resistance.^[8]^ Such hybrids were created by joining various biologically active agents into a single heteromeric unit with the aim of retaining the pharmacological actions of each component.^[9]^ The aim of this research project is to link two biologically active linear polyamines by an appropriately chosen/designed linker (Figure 1). This approach might improve their affinity and potency. Furthermore, the designed homo‐ (**14** and **15**) and hetero‐ (**16**) dimeric linear polyamine amides (Figure 1) have additional amino functional groups (cationic groups) that may increase the antibiofilm activity.

Reacting amino functional groups in linear polyamines with three or five equivalents of acrylonitrile is another method to increase the number of amino functional groups (positive charges), from three amines (in norspermidine, 3.3) to six (in compound **22**) or eight amines (in compound **23**) after reducing the nitrile functional groups to their corresponding amino groups. The designed branched polyamines **22** and **23** (Figure 1) will be tested microbiologically and compared to homo‐ (**14** and **15**) and hetero‐ (**16**) linear dimeric polyamine amides.

## Results and Discussion

### Selective Protection of the Reactive Amines on Linear Polyamines

**Scheme 1:**
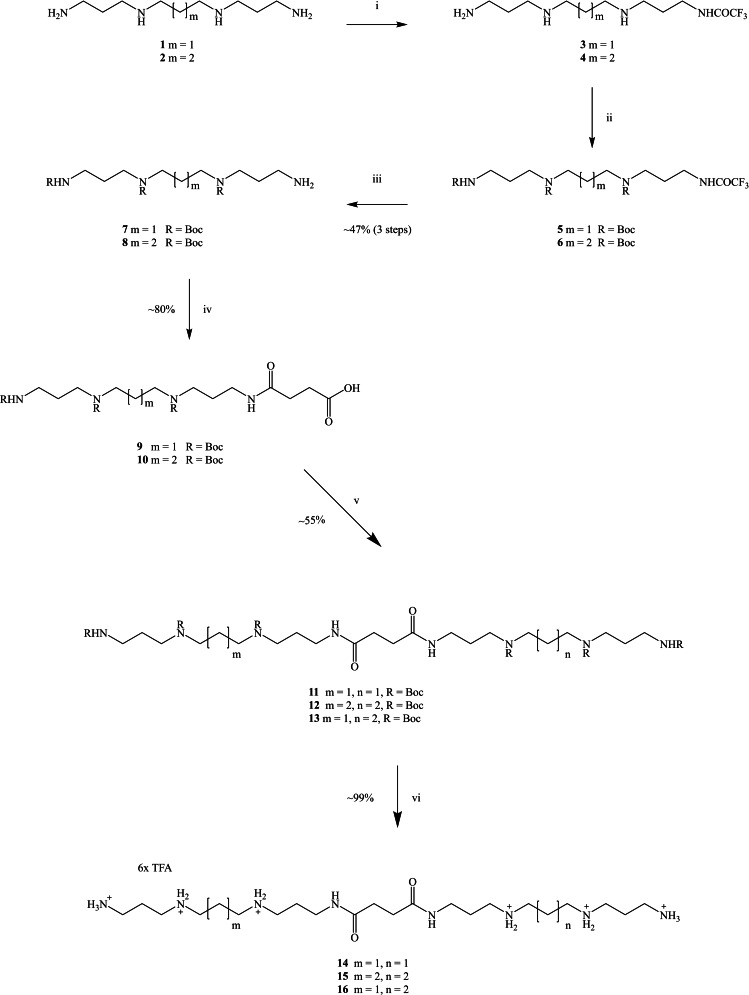
*Reagents and conditions*: (i) EtOOCCF_3_, MeOH, −78 °C, 18 h; (ii) Boc_2_O, MeOH, 0 °C, 18 h; (iii) aq. NH_3_, 20 °C, 18 h; (iv) succinic anhydride, anhydrous pyridine, 20 °C, 18 h; (v) HBTu, R‐NH_2_ (**7** or **8**), TEA, anhydrous DMF, 20 °C, 18 h; (vi) DCM/TFA (9 : 1 *v*/*v*), 20 °C, 18 h.

The ability to link polyamines requires selective protection, in order to avoid unwanted side products. However, the protection of three amines out of four is always low‐yielding and needs chromatographic purification. Alternatively, mono‐protection using benzyl chloroformate (CbzCl) or di‐*tert*‐butyldicarbonate (Boc anhydride) either results in a low yield, which is not practical, or requires a long time for chromatographic purification. We have previously reported^[10]^ that using trifluoroacetyl as a protecting group for one amine out of four can be controlled by decreasing the temperature and the concentration. Subsequent removal under basic conditions makes trifluoroacetyl an ideal protecting group for preparing unsymmetrical polyamine amides. The ratio between −NH_2_ and ethyl trifluoroacetate (source of the protecting group) is important not only to avoid protection of both primary amines −NH_2_), but also to avoid protection of primary (−NH_2_) and secondary amines (−NH−). The introduction of trifluoroacetyl as a protecting group will be favoured for primary amines over secondary amines because the secondary amino groups on **1** and **2** are more sterically hindered than the primary amino groups. Taking all these advantages into consideration, trifluoroacetyl as a protecting group makes is superior compared to CbzCl and di‐*tert*‐butyl dicarbonate ((Boc)_2_O) for the purpose of gram‐scale protection of polyamines. Thus, using this method, triBoc **7** and **8** were synthesised by first protecting one amine using ethyl trifluoroacetate, then Boc protecting the other groups, before finally revealing the first amine by trifluoroacetyl removal (Scheme 1). TriBoc **7** and **8** were synthesised by the addition of one equivalent of ethyl trifluoroacetate to a methanolic solution of starting materials **1** and **2** at −78 °C to obtain mono‐trifluoroacetamides **3** and **4**, respectively. At this point, analysis by mass spectrometry showed **3** and **4** with the correct mass (HRMS: found 285.1810, C_11_H_24_F_3_N_4_O requires 285.1824 [M+H]^+^ for compound **3**; HRMS: found 299.1971, C_12_H_26_F_3_N_4_O requires 299.1980 [M+H]^+^ for compound **4**). The products were not isolated. Instead, in the same methanolic solution, three equivalents of di‐*tert*‐butyldicarbonate were added to afford fully protected polyamines **5** and **6** (HRMS: found 585.3411, C_26_H_48_F_3_N_4_O_7_ requires 585.3397 [M+H]^+^ for compound **5**; HRMS: found 621.3546, C_27_H_49_NaF_3_N_4_O_7_ requires 621.3553 [M+Na]^+^ for compound **6**). The trifluoroacetamide group was selectively removed by increasing the pH to above 11 with concentrated aqueous ammonia to afford polyamines **7** and **8**, both with unmasked primary amino groups (Scheme 1). The spectral data obtained for compounds **7** and **8** agree with those we have reported previously.^[10]^

### Synthesis of Polyamine Carboxylic Acid Analogues

For possible application as biofilm disruptors, the aim is to link two active pharmacophores in order to increase their activity in preventing the formation of the biofilm, and/or destroying the existing biofilm.[^1^, ^2^, ^3^, ^4^, ^8^, ^10^] Succinic anhydride was selected to link two of the same or different polyamine biofilm disruptors. Succinic anhydride is reactive and well suited to introduce a short (four carbon atoms) spacer and a useful carboxylic acid functional group from which amides may be obtained (e. g., from the same or different linear polyamines).^[11]^ Compounds **9** and **10** were synthesised by the addition of one equivalent of succinic anhydride to a solution of triBoc **7** and **8** in anhydrous pyridine at 20 °C (Scheme 1). All spectral data confirmed that reactions took place successfully. The 1H NMR spectrum of compound **10** shows two triplets at 2.45 and 2.58 ppm, representing the two CH_2_ groups between the amide group and the carboxylic acid in the short linker. The carbon signals at 173.4 and 174.8 ppm in the ^13^C NMR spectrum are associated with the carbon atoms of the carboxylic acid and the amide group, respectively. Moreover, ^1^H‐^13^C cross peaks in the HMBC NMR spectrum between the two triplets and the two carbon signals were observed (Figure S1, Supporting Information). Mass spectrometry showed correct masses for compounds **9** and **10** (HRMS: found 587.3730 (*m/z*), C_28_H_51_N_4_O_9_ requires 587.3734 (*m/z*) [M−H]^−^ for compound **9**; HRMS: found 601.3867 (*m/z*), C_29_H_53_N_4_O_9_ requires 601.3860 (*m/z*) [M−H]^−^ for compound **10**).

### Synthesis of Homo‐ and Hetero‐dimeric Linear Polyamines

Carboxylic acids **9** and **10** were separately coupled to mono‐amines **7** and **8**, respectively, to obtain the corresponding target homo‐dimeric polyamines **11** and **12**. Compound **13** was synthesised in order to have two different polyamines linked together (in a hetero‐dimeric linear polyamine) by coupling carboxylic acid **9** to triBoc **8**. Compounds **11**, **12**, and **13** were synthesised in good yield after trying various coupling regents under different experimental conditions, (e. g., DCC/DMAP in anhydrous DMF or anhydrous DCM at 20 °C, and EDC⋅HCl/HOBT in anhydrous DMF) with low yields of about 5–10 %. A satisfactory (practical) yield was obtained by the addition of one equivalent of HBTu (as a more reactive carboxylic activating agent) to a solution of **14**, **15**, and **16**, respectively, in anhydrous DMF at 20 °C, followed by the addition of one equivalent of the amines **7** or **8**. ^1^H NMR spectra show a singlet at 2.52 ppm integrating for four protons with hetero‐dimeric polyamines. This singlet represents the two CH_2_ groups between the two new amide functional groups (RNHCO**CH_2_CH_2_** CONHR) instead of two triplets, as shown in compounds **11**, **12**, and **13** (Figure S2), as the magnetic environment of the two CH_2_ groups between the amide groups (RNHCO**CH_2_CH_2_** CONHR) is similar. Only one carbonyl signal was observed at ≈175 ppm with a higher intensity, relating to both amide carbonyl groups, while the carboxylic acid signal in the ^13^C NMR spectrum had correspondingly disappeared. Removal of the Boc protecting groups in compounds **11**, **12**, and **13** proceeded smoothly using trifluoracetic acid (TFA) in DCM (1 : 9 *v*/*v*) stirred for 18 h at 20 °C, which was then then concentrated *in vacuo* and lyophilised to yield the desired hetero‐dimeric linear polyamines **14**, **15**, and **16** as their poly‐TFA salts confirmed by mass spectrometry as well as by IR and NMR spectroscopy.

### NMR Spectroscopic Structural Assignments for Homo‐ (14 and 15) and Hetero‐ (16) Dimeric Linear Polyamines

All ^1^H and ^13^C atom signals of compounds **14**, **15**, and **16** were unambiguously assigned using 2D ^1^H‐^13^C HSQC and ^1^H‐^13^C HMBC NMR spectroscopy. The ^1^H NMR magnetic resonance of the methylene groups of poly‐TFA salts of compounds **14**, **15**, and **16** are observed in four distinct regions. Around 3.3 ppm, the methylene groups adjacent to amide groups (11‐CH_2_ for **14**, 12‐CH_2_ for **15**, 11‐CH_2_, 18‐CH_2_ for **16**) are found. At 3.0–3.2 ppm, methylene groups adjacent to primary and secondary amino groups resonate (10×NCH_2_), while the two methylene groups of the linker (RNHCO**CH_2_CH_2_** CONHR) appear around 2.5 ppm. At 1.7–1.8 ppm, the methylene groups separated from NH_2_, NH, or CONH by one CH_2_ group on each side (2‐CH_2_, 6‐CH_2_, 10‐CH_2_ for compound **14**, 2‐CH_2_, 11‐CH_2_ for **15**, 2‐CH_2_, 6‐CH_2_, 10‐CH_2_, 19‐CH_2_, 28‐CH_2_ for **16**) appear, while methylene groups separated from amine and amide groups (NH_2_, NH, or CONH) by one CH_2_ group on one side and two from another side (6‐CH_2_, 7‐CH_2_ for compound **15** and 23‐CH_2_, 24‐CH_2_ for **16**) resonate around 1.7 ppm. Methylene groups in α position to an amide (11‐CH_2_ for compound **14**, 12‐CH_2_ for **15**, 11‐CH_2_, 18‐CH_2_ for compounds **16**) are more de‐shielded, and therefore have the highest chemical shift. The protonation of primary and secondary amine functional groups causes a de‐shielding of the methylene functional group α to the nitrogen atom, causing a downfield shift by ≈1 ppm.^[9]^ Therefore, CH_2_ groups located next to an amine are more de‐shielded than those located further away, (1‐CH_2_, 3‐CH_2_, 5‐CH_2_, 7‐CH_2_, 9‐CH_2_ for **14**, 1‐CH_2_, 3‐CH_2,_ 5‐CH_2_, 8‐CH_2,_ 10‐CH_2_ for **15**, and 1‐CH_2_, 3‐CH_2_, 5‐CH_2_, 7‐CH_2_, 9‐CH_2_, 20‐CH_2_, 22‐CH_2_, 25‐CH_2_, 27‐CH_2_, 29‐CH_2_ for **16**).

**Figure 2:**
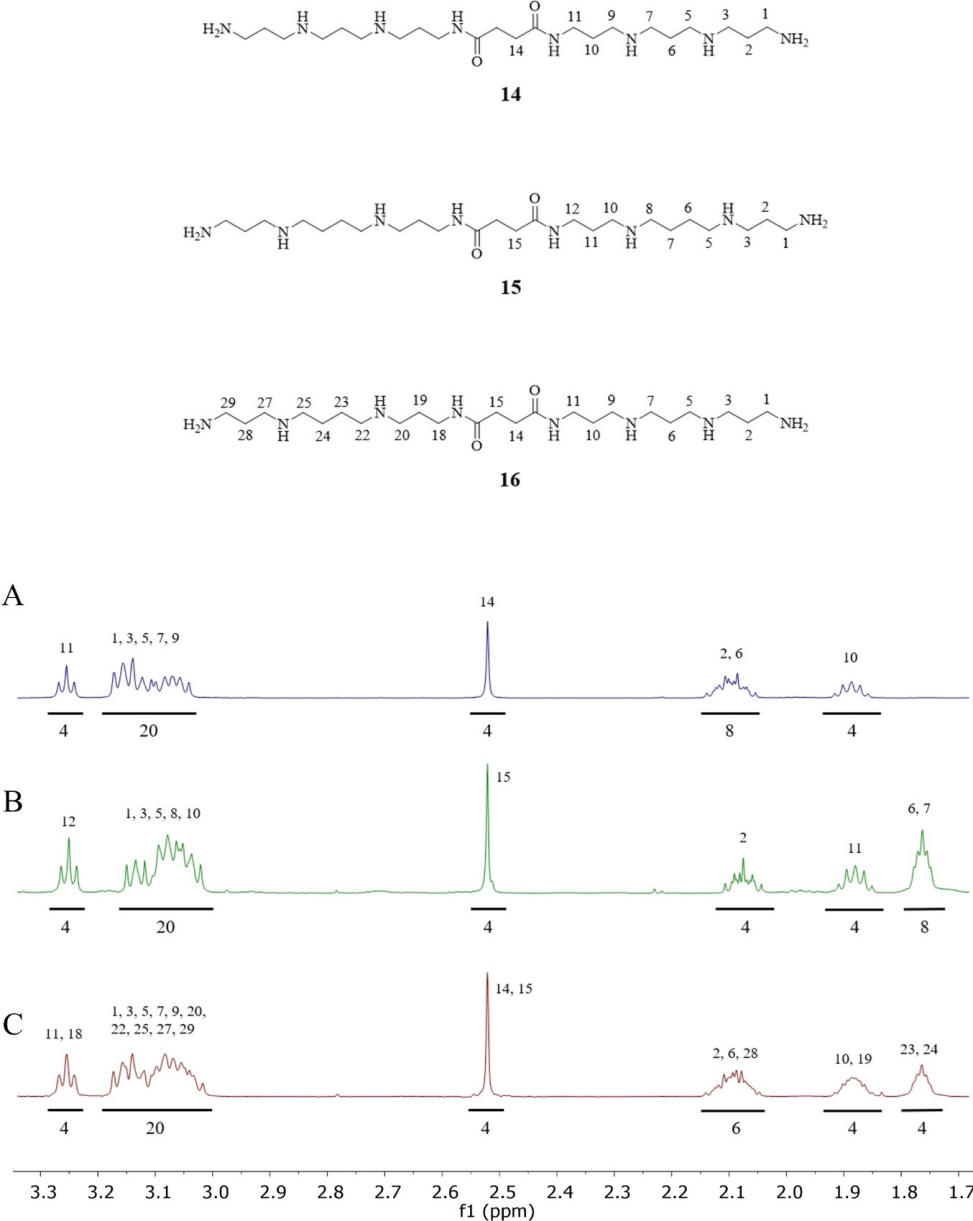
The ^1^H NMR spectra of compounds (A) **14**, (B) **15**, and (C) **16** as their respective TFA salts, referenced to the residual solvent peak (HOD) at 4.79 ppm in 99.7 % D_2_O at 25 °C.

Methylene groups in β positions to amide and secondary amines (10‐CH_2_ for compound **14**, 11‐CH_2_ for compound **15**, and 10‐CH_2_, 19‐CH_2_ for compound **16**) are less de‐shielded, and therefore have smaller chemical shifts than methylene groups β to primary and secondary or secondary and secondary amines (2‐CH_2_, 6‐CH_2_ for compound **14**, 2‐CH_2_ for compound **15**, and 2‐CH_2_, 6‐CH_2_, 28‐CH_2_ for compound **16**). However, protons 6‐CH_2_, 7‐CH_2_ for compound **15** and 23‐CH_2_, 24‐CH_2_ for compound **16** are in both β and γ positions to secondary amines, and for this reason, their ^1^H NMR signals are shifted slightly upfield by ≈0.2 ppm compared to methylene groups in β positions to amide and secondary amines (Figure 2).

The ^13^C NMR signals of methylene groups of poly‐TFA salts of compounds **14**, **15**, and **16** can be found in four distinct regions. Around 175 ppm, signals of the amide groups (2× NHCO for compounds **14**, **15**, and **16**) are found; around 35–50 ppm, the methylene groups adjacent to primary amines, secondary amines and amid groups (12× NCH_2_) resonate. The two methylene groups of the linker (RNHCO **CH_2_ CH_2_** CONHR) resonate around 30 ppm, while, around 20–30 ppm, signals for the methylene groups separated from amide, and secondary amino groups or separated from amines (NH_2_ or/and NH) by one CH_2_ group on each side (2‐CH_2_, 6‐CH_2_, 10‐CH_2_ for compound **14**, 2‐CH_2_, 11‐CH_2_ for compound **15**, 2‐CH_2,_ 6‐CH_2_, 10‐CH_2_, 19‐CH_2_, 28‐CH_2_ for compound **16**), or methylene groups separated from the secondary amines by one CH_2_ functional group on one side and two CH_2_ groups on the other side (6‐CH_2_, 7‐CH_2_ for compound **15**, and 23‐CH_2_, 24‐CH_2_ for compound **16**) can be found.

### Branched Polyamines 22 and 23

The aim of designing and synthesising compounds **18** and **19** was to reduce the three nitrile functional groups to the corresponding primary amines **22** and **23** and to then compare their biological activity against the biological activity of linear polyamines **14**, **15**, and **16**.

The reduction of these compounds following a reported method using Raney nickel as a catalyst and sodium hydroxide (co‐catalyst) under a hydrogen pressure of 2.7 bar[^12^, ^13^] for the reduction of compounds **18** and **19** was successful. However, due to the high polarity of the corresponding products **22** and **23**, the branched polyamines could not be extracted from the aqueous sodium hydroxide solution with a chloroform:methanol (85: 15 *v*/*v*) mixture. Therefore, the primary amines were protected using the commercially available Boc_2_O protecting group. The purpose of protecting all amino functional groups on compound **22** and **23** was to decrease the polarity to allow for easy extraction from the aqueous sodium hydroxide solution and hence purification.

**Scheme 2:**
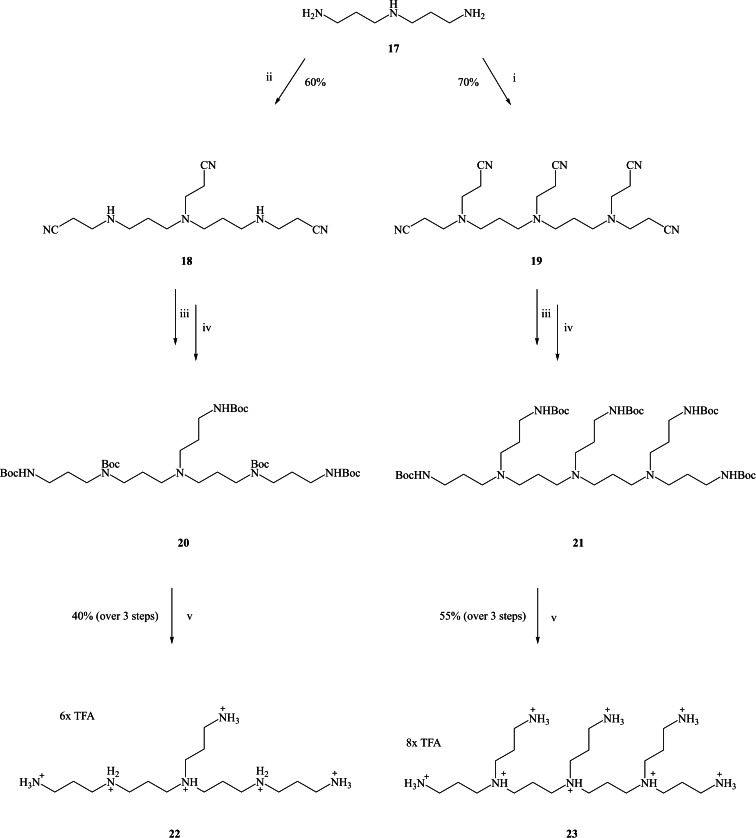
*Reagents and conditions*: (i) acrylonitrile (5 equiv.), EtOH, 20 °C, 18 h; (ii) acrylonitrile (3 equiv.), EtOH, 20 °C, 18 h; (iii) Raney nickel, H_2_, NaOH, EtOH, 20 °C, 18 h; (iv) Boc_2_O, MeOH, 20 °C, 18 h; (v) DCM/TFA (9 : 1 *v*/*v*), 20 °C, 18 h.

The commercially available norspermidine **17** was reacted with three or five equivalents of acrylonitrile in EtOH at 20 °C to undergo a 1,4‐Michael addition reaction to obtain compounds **18** and **19**, respectively, isolated in 60–70 % yield after column chromatography (Scheme 2). Their spectral data agree with those reported previously.^[14]^ The IR spectrum confirmed the presence of nitrile (CN) functional group in the desired derivatives **18** and **19** with a sharp band at ≈2253 cm^−1^. Mass spectrometry showed the correct mass. Moreover, ^13^C NMR spectroscopy showed a low intense signal at 118.8 ppm that assigned to nitrile carbon. The catalytic hydrogenation of nitrile compounds **18** and **19** was more difficult than expected. In order to reduce the nitrile groups, different (catalytic) hydrogenation methods were tried. Compounds **18** and **19** were dissolved in methanol, Pd/C (10 %) was used as a catalyst, and a hydrogen balloon as a source of H_2_,^[15]^ but TLC showed a major spot of the starting material. When Raney nickel was used instead of Pd/C (10 %), the reaction was found not successful as, again, the TLC showed a major spot of the starting material.^[16]^ LiAlH_4_ has been used to reduce nitrile functional groups to primary amines.^[17]^ Compound **2** was dissolved in anhydrous tetrahydrofuran (THF) and then slowly added to the mixture. The reaction mixture was stirred for a further 18 h under an atmosphere of anhydrous nitrogen. TLC showed a mixture of several spots which were deemed to difficult to be purified to homogeneity.

However, another method had been reported to reduce nitriles to primary amines by using Raney nickel (catalyst) and sodium hydroxide (co‐catalyst) under a hydrogen pressure of 2.7 bar.[^12^, ^13^] Following this method, at a reduced 1 bar of hydrogen pressure, the reaction was successful. Sodium hydroxide was dissolved in 20 mL of EtOH and added to the mixture of compounds **18** or **19** and Raney nickel. The atmosphere over the solution was evacuated and replaced with N_2_ gas three times, and then replaced with H_2_. The solution was stirred under H_2_ for a further 18 h at 20 °C. The solution mixture was then filtered through Celite. Without further purification, an excess of (Boc)_2_O was added to the ethanolic solution. Compounds **20** and **21** were extracted with chloroform in order to remove the NaOH. Without further analysis, removal of the Boc protecting groups in compounds **20** and **21** proceeded smoothly using trifluoracetic acid (TFA) in dichloromethane (DCM) (1 : 9 *v*/*v*) stirred for 18 h, then concentrated *in vacuo* and lyophilised to yield the desired branched polyamines **22** and **23** as poly‐TFA salts confirmed by mass spectrometry, and IR spectroscopy. The ^13^C NMR spectra showed the disappearance of the nitrile peaks at 118.9 and 119.2 ppm for compound **18** and at 118.8 and 119.5 ppm for compound **19**.

### NMR Spectroscopic Structural Assignments for Compounds 18 and 19

**Figure 3:**
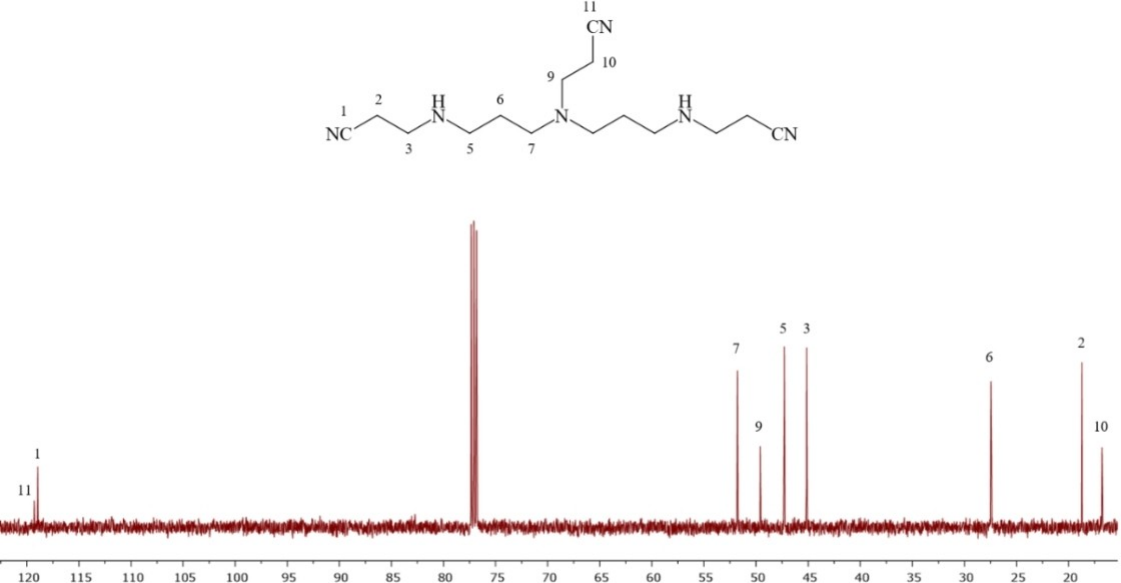
The ^13^C NMR spectrum of symmetrical **18** referenced to TMS at 0.0 ppm in 99.7 % CDCl_3_ at 25 °C.

The ^13^C NMR spectrum of compound **18** shows nine peaks, of which the two low‐intensity downfield peaks resonating at 119.2 and 118.9 ppm are assigned to nitrile carbons 11‐CN and 1‐CN, respectively. However, their peaks intensities are different. The signal intensity of carbon resonating at 118.9 ppm is significantly higher than that resonating at 119.2 ppm, and this intensity difference formed the basis for the assignments of the nitrile carbon atoms (11‐CN and 1‐CN; Figure 3). The next four carbon peaks resonating at 51.7, 49.5, 47.2 and 45.1 ppm are assigned for N‐CH_2_ carbons, 7‐CH_2_, 9‐CH_2_, 5‐CH_2_ and 3‐CH_2_, respectively, and are de‐shielded due to the inductive effect of nitrogen. Nevertheless, among these four carbon peaks, the assignment for 9‐CH_2_ at 49.5 ppm is the only secured one due to its lower relative intensity.

The next three carbon peaks resonating at 27.4, 18.7, and 16.7 ppm are assigned to carbon atoms 6‐CH_2_, 2‐CH_2_ and 10‐CH_2_, respectively. 2‐CH_2_ and 10‐CH_2_ have a similar chemical environment, however, the carbon peak resonating at 16.7 ppm is securely assigned to 10‐CH_2_ due to its lower relative intensity compared to the carbon peaks at 18.7 ppm, that are assigned for the equivalent carbons 2‐CH_2_. For this reason, the carbon peak at 16.7 ppm is assigned to 10‐CH_2_ and the carbon peak at 18.7 ppm is assigned to 2‐CH_2_.

Compound **18** has been reported and synthesised previously to make neurotoxins.^[14]^ Balczewski et al. reported the ^13^C NMR spectral data for compound **18** with ten carbons, therefore they reported an extra carbon signal. In its chemical structure, compound **18** has 15 carbon atoms and a symmetrical centre. Six carbon atoms, which are 1‐CH_2_, 2‐CH_2_, 3‐CH_2_, 5‐CH_2_, 6‐CH_2_, and 7‐CH_2_, have an equivalent carbon atom associated to them. The three carbons left (9‐CH_2_, 10‐CH_2_, and 11‐CH_2_) have no equivalent carbon atom (Figure 3). For this reason, the ^13^C NMR spectrum of **18** shows nine peaks.

In contrast, compound **19** has three symmetrical centres, nitrogen N‐8 and the two N‐4 nitrogen atoms. Therefore, the ^13^C NMR spectrum of compound **19** (also) shows nine peaks, of which the two low‐intensity downfield peaks at 119.5 ppm and 118.8 ppm are assigned to nitrile carbon 11‐CN and the four equivalent nitrile carbons 1‐CN, due to the higher relative intensity, respectively.

**Figure 4:**
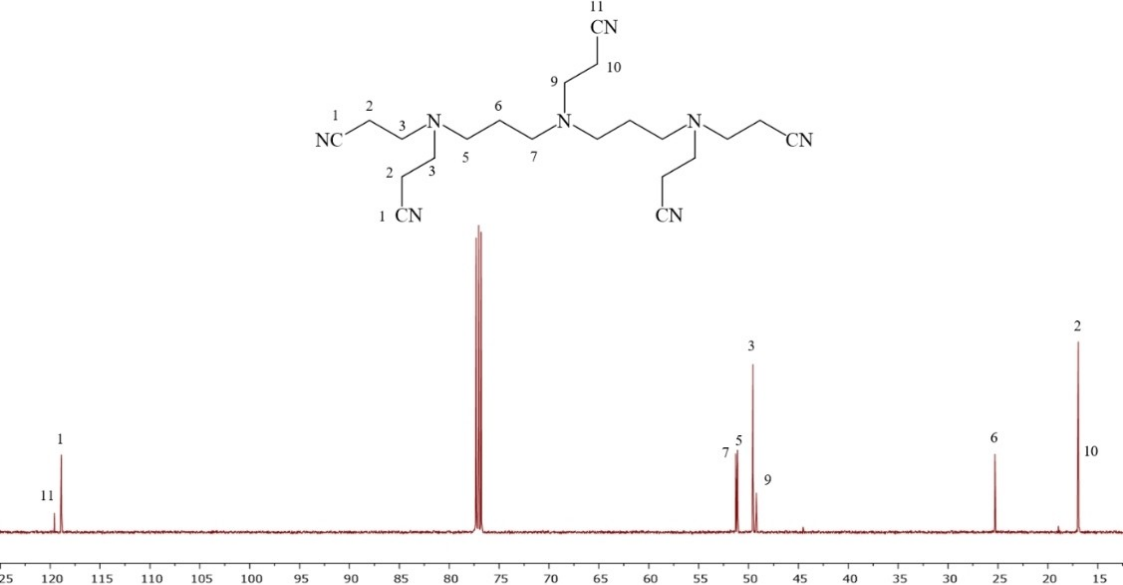
The ^13^C NMR spectrum of symmetrical **19** referenced to TMS at 0.0 ppm in 99.7 % CDCl_3_ at 25 °C.

The next four peaks at 51.2, 51.1, 49.5, and 49.2 ppm are assigned to N‐CH_2_ carbons, that is, 7‐CH_2_, 5‐CH_2_, 3‐CH_2_, and 9‐CH_2_, respectively, that are de‐shielded due to the inductive effect of nitrogen. There is a small chemical shift difference (≈0.1 ppm) between the carbon atoms 5‐CH_2_ and 7‐CH_2_ due to the presence of different numbers of alkyl and CN substituents. Therefore, the peak at 51.3 ppm is assigned to the two equivalent carbons 5‐CH_2_ due to the presence of two δ‐CN and one δ‐alkyl substituent, while the peak at 51.2 ppm is assigned to the two equivalent carbon atoms 7‐CH_2_ due to the presence of one δ‐CN and three δ‐alkyl substituents. However, due to the very similar chemical shifts of these carbon atoms, this assignment is more tentative. The peaks at 49.6, 25.4, and 17.0 ppm are assigned to 3‐CH_2_ (four equivalent carbon atoms), 6‐CH_2_ (two equivalent carbon atoms), 2‐CH_2_ (four equivalent carbon atoms), respectively, due to their higher relative intensity, while the signals at 49.3 and 16.9 ppm are assigned to 9‐CH_2_ and 10‐CH_2_, respectively, due to their lower relative intensity (Figure 4).

### Antimicrobial Testing of Target Compounds Synthesized

None of the short, linear, naturally occurring polyamines, norspermidine, spermidine, thermine, spermine, is an antibiotic (MIC >256 μg mL^−1^) and our succinimide‐linked short linear polyamine amides are likewise not microbiologically active, displaying MIC and MBIC values >64 μg mL^−1^ on MSSA and PAO1. However, increasing the number of positive charges from 3, 4, 6 to 8 in compound **23** showed a 50 % reduction in the biofilm of PAO1 at 16–32 μg mL^−1^. This is a positive result, achieved at a concentration comparable with those results for a dithiourea diamine at 64 μg mL^−1[3]^ and a tetramine being a long lipid amide of norspermidine, capped by acylation at both ends with lysine, therefore possessing four positive charges and displaying antibiofilm activity at 60 μg mL^−1^.^[5]^

## Conclusions

We have described the practical synthesis of a series of substituted polyamines and polyamine amides of succinic acid. These compounds do not display antibacterial activity against Gram‐positive or Gram‐negative bacteria, including MRSA and *Pseudomonas aeruginosa*. However, the most charged analogue **23**, a branched polyamine carrying eight positive charges at physiological pH, displays antibiofilm activity with a 50 % reduction in PAO1 at 16–32 μg mL^−1^.

## Experimental Section

### General Methods

**Chemicals and Materials**: CDCl_3_, CD_3_OD, and D_2_O were purchased from Goss Scientific (UK). Aqueous ammonia (32 %), dichloromethane (DCM), dimethylformamide (DMF), 1,4‐dioxane, ethanol, ethyl acetate, methanol, and triethylamine (TEA) were purchased from VWR (UK). Anhydrous dimethylformamide (DMF) and anhydrous pyridine were purchased from Fisher Scientific (UK). Norspermine **1**, spermine **2**, norspermidine **25**, acrylonitrile, anhydrous potassium bromide (KBr), anhydrous sodium sulfate (Na_2_SO_4_), ethyl trifluoroacetate, Raney®‐Nickel, ninhydrin, sodium hydroxide, di‐*tert*‐butyl dicarbonate ((Boc)_2_O), succinic anhydride, *N,N,N′,N′*‐tetramethyl‐*O*‐(1*H*‐benzotriazol‐1‐yl)uronium hexafluorophosphate (HBTu), and trifluoroacetic acid (TFA) were purchased from Sigma‐Aldrich (UK).

Column chromatography was performed over silica gel 60–120 mesh (purchased from Sigma‐Aldrich, UK) using different ratios of aqueous ammonia (32 %), DCM, ethanol, ethyl acetate, and methanol as eluents.

Thin‐Layer Chromatography (TLC) over silica gel was performed using aluminium‐backed sheets coated with Kieselgel 60 F_254_ purchased from Merck (UK). Ninhydrin TLC spray reagent, ninhydrin (0.2 g) in ethanol (100 mL), was used for detecting amine functional groups.

**Instrumentation**: NMR spectra including ^1^H, ^13^C, heteronuclear single quantum correlation (HSQC) and heteronuclear multiple bond correlation (HMBC) were recorded on Bruker Avance III (operating at 500.13 MHz for ^1^H and 125.77 MHz for ^13^C) spectrometers at 25 °C. MestReNova was used for processing the spectra. ^1^H and ^13^C chemical shifts (δ) were observed and are reported in parts per million (ppm) relative to tetramethylsilane (TMS) at 0.00 ppm as an internal reference or residual solvent peaks, HDO at 4.79 ppm and using the intrinsic lock signal for ^13^C NMR. High Resolution Time‐of Flight (HR‐TOF) mass spectra were obtained on a Bruker Daltonics “micrOTOF” mass spectrometer using electrospray ionisation (ESI) (loop injection +ve ion mode).

**Bacterial strains, culture conditions, and minimum inhibitory concentration (MIC) determination**: Bacterial strains used in this study are *Staphylococcus aureus* grown on tryptic soy agar (TSA; Sigma‐Aldrich) for 18 h at 37 °C, and *Pseudomonas aeruginosa* grown on Luria‐Bertani agar (LBA; Sigma‐Aldrich) for 18 h at 37 °C. The minimum inhibitory concentration (MIC) of polyamines against these bacterial species was determined using the broth microdilution method as described by the Clinical and Laboratory Standards Institute (CLSI).^[18]^ Individual pure colonies of the above bacterial species were used to inoculate separate 15 mL polystyrene test tubes (ThermoFisher) containing 3 mL of cation‐adjusted Mueller‐Hinton broth (MHB; Oxoid). Growth agar and broth were prepated according to the manufacturer's instructions. Bacterial cultures were incubated for 18 h at 37 °C with shaking at 180 rpm (New Brunswick Innova 44/R incubator). The 18 h bacterial cultures were subsequently diluted 1 : 100 in fresh MHB and cultured at 37 °C with shaking at 180 rpm to exponential phase of growth, defined as reaching an absorbance (OD_600nm_) within the range of 0.5–0.6. Absorbance was measured using a 1 mm cuvette and DS‐11 Spectrophotometer (DeNovix). Polyamines were reconstituted in sterile deionised water, then diluted in MHB and dispensed into a 96‐well round bottom microtiter plate (Costar) to a final concentration range of 256–0.125 μg mL^−1^. Aliquots of 0.5 McFarland standardized inoculum of bacteria were dispensed into wells containing polyamines to a final inoculum of 5×10^5^ CFU mL^−1^ with a no‐compound control. Bacterial cultures were grown statically at 37 °C for 24 h (ThermoScientific HeraTherm). The MIC was defined as the lowest concentration of compound to result in no visible growth measured through inspection of turbidity.

**General Procedure Boc removal**: A solution of Boc protected polyamine in DCM (9 mL) was deprotected by adding TFA (1 mL) at 20 °C. The solution was stirred for 18 h, then concentrated *in vacuo* and lyophilised to yield the desired product poly‐TFA salt as a pale yellow viscous oil.

### Supporting Information Summary

Synthesis procedures, purification to homogeneity and HR‐MS and NMR spectroscopic assignments of linear polyamines and their conjugates **7**–**16**, **18**, **19**, and branched polyamines **22** and **23** are given in the Supporting Information.

## Conflict of interest

The authors declare no conflict of interest.

1

## Supporting information

As a service to our authors and readers, this journal provides supporting information supplied by the authors. Such materials are peer reviewed and may be re‐organized for online delivery, but are not copy‐edited or typeset. Technical support issues arising from supporting information (other than missing files) should be addressed to the authors.

Supporting Information

## Data Availability

The data that support the findings of this study are available in the supplementary material of this article.
