# Supplementary material for: Practical Synthesis of Polyamine Succinamides and Branched Polyamines
Source: ChemistryOpen. 2022 Oct 25;11(10):e202200147. doi: 10.1002/open.202200147 (PMC9596609; doi:10.1002/open.202200147)
Supplement: Supplementary file 1 — Supporting Information [file OPEN-11-e202200147-s001.pdf]

ChemistryOpen

Supporting Information

Practical Synthesis of Polyamine Succinamides and Branched Polyamines

Abdulaziz H. Alkhzem, Maisem Laabei, Timothy J. Woodman, and Ian S. Blagbrough*

(N^1,N^4,N^8-Tri-*tert*-butoxycarbonyl)-1,11-diamino-4,8-diazaundecane 7

A solution of norspermine (thermine) **1** (2.00 g, 10.6 mmol) in methanol (150 mL), at -78 °C under nitrogen, was treated with ethyl trifluoroacetate (1.51 g, 10.6 mmol, 1 equiv.) dropwise over 15 min. Stirring was continued for a further 45 min, then the temperature was increased to 20 °C for 18 h to afford the mono-trifluoroacetamide **3**. Without purification, the remaining amino functional groups were protected using di-*tert*-butyldicarbonate (6.95 g, 31.8 mmol, 3.0 equiv.) in methanol (20 ml) at 0 °C over 10 min. The reaction was then warmed to 20 °C and stirred for a further 18 h to afford the fully protected polyamine **5**. The trifluoroacetate protecting group was then removed by increasing the pH of the solution to above 11 with conc. aq. ammonia (32%) and then stirring at 20 °C for 18 h. The solution was concentrated under reduced pressure. The crude product was purified by column chromatography with DCM in MeOH (9.5:0.5 v/v). After combining fractions and concentrating them, the desired product **7** was obtained as a colourless oil (2.50 g, 48%). TLC analysis showed one spot (R_f = 0.5, DCM: MeOH: aq. ammonia (32%), 70:10:1 v/v/v). HRMS: Found 489.3641 (*m/z*), $C_{24}H_{49}N_4O_6$ requires 489.3573 (*m/z*) $[M + H]^+$; IR (film); 1689 (C=O) cm^{-1}; ^1H NMR, 500 MHz ($CDCl_3$): 1.37-1.51 (m, 27H, 9 x CH_3 Boc), 1.66-1.78 (m, 4H, 2-CH_2, 6-CH_2, overlapping), 1.88-193 (m, 2H, 10-CH_2), 2.91-2.98 (t, J = 6.4 Hz, 2H, 11-CH_2), 3.05-3.38 (m, 10H, 1-CH_2, 3-CH_2, 5-CH_2, 7-CH_2, 9-CH_2); ^{13}C NMR, 125.77 MHz ($CDCl_3$): 25.9 (10-CH_2), 27.4, 27.8, 29.0 (9 x CH_3 Boc 2-CH_2, 6-CH_2, overlapping), 36.5 (11-CH_2), 40.6, 40.9, 46.1, 46.4 (1-CH_2, 3-CH_2, 5-CH_2, 7-CH_2, 9-CH_2), 79.9-80.8 (3 x Cq Boc), 156.5-158.0 (3 x C=O Boc).

(N^1,N^4,N^9-Tri-*tert*-butoxycarbonyl)-1,12-diamino-4,9-diazadodecane 8

A solution of spermine **2** (0.50 g, 2.47 mmol) in methanol (100 ml), at -78 °C under nitrogen, was treated with ethyl trifluoroacetate (0.35 g, 2.47 mmol, 1 equiv.). The ethyl trifluoroacetate was added dropwise over 20 min. stirring was continued for a further 30 min, then the temperature was increased to 20 °C to afford predominantly the mono-trifluoroacetamide **4**. Without purification, the remaining amino functional groups were protected using di-*tert*-butyldicarbonate (1.61 g, 7.41 mmol, 3.0 equiv.) in methanol (15 ml) over 10 min. The reaction was then warmed to 20 °C and stirred for a further 18 h

to afford the fully protected polyamine **6**. The trifluoroacetate protecting group was then removed by increasing the pH of the solution to above 11 with conc. aq. ammonia (32%) and then stirring at 20 °C for 18 h. The solution was concentrated under reduced pressure. The column chromatography was elution with DCM in MeOH (9.5:05 v/v). After combining fractions and concentrating them, the desired product **8** was obtained as a colourless oil (0.57 g, 46%). TLC analysis showed one spot (R_f = 0.6, DCM:MeOH: aq. ammonia (32%), 50:10:1 v/v/v). HRMS: Found 503.3728 (*m/z*), $C_{25}H_{51}N_4O_6$ requires 503.3730 (*m/z*) $[M + H]^+$; IR (film); 1692 (C=O) cm^{-1}; ^{1}H NMR, 500 MHz ($CDCl_3$): 1.41-1.53 (m, 31H, 9 x CH_3 Boc, 6-CH_2, 7-CH_2, overlapping), 1.60-1.65 (m, 4H, 2-CH_2, 11-CH_2), 2.70 (t, *J* = 6.7 Hz, 2H, 12-CH_2), 3.04-3.29 (m, 10H, 1-CH_2, 3-CH_2, 5-CH_2, 8-CH_2, 10-CH_2, overlapping); ^{13}C NMR, 125.77 MHz ($CDCl_3$): 25.5, 25.8 (6-CH_2, 7-CH_2), 28.4, 29.0, 34.0 (9 x CH_3 Boc, 2-CH_2, 11-CH_2, overlapping), 37.3 (12-CH_2), 42.0, 42.2, 43.9, 44.1 (1-CH_2, 3-CH_2, 5-CH_2, 8-CH_2, 10-CH_2, overlapping), 79.2-81.4 (3 x Cq Boc), 156.1-157.9 (3 x C=O Boc).

Synthesis of compound 9

A solution of the tri-Boc protected norspermine (thermine) **7** (0.35 g, 0.71 mmol) in anhydrous pyridine (5 mL) under nitrogen was treated with succinic anhydride (0.07 g, 0.71 mmol, 1 equiv.) at 20 °C. The solution was stirred for a further 18 h. The solution was then concentrated *in vacuo*, and the crude material was extracted with chloroform (3 x 15 mL). The combined organic extracts were dried (Na_2SO_4), filtered, and concentrated in *vacuo*. The desired product **9** was obtained as a colourless oil (0.31 g, 75%). TLC analysis showed one spot (R_f = 0.3 (EtOAc: ethanol: aq. ammonia (32%); 7:2:1 v/v/v). HRMS: Found 587.3730 (*m/z*), $C_{28}H_{51}N_4O_9$ requires 587.3734 (*m/z*) $[M - H]^-$; IR (film); 3746-3074 (COOH) and 1629 (C=O), cm^{-1}; ^{1}H NMR, 500 MHz (CD_3OD): 1.42-1.48 (m, 27H, 9 x CH_3 Boc), 1.64-1.80 (m, 6H, 2-CH_2, 6-CH_2, 10-CH_2, overlapping), 2.51 (t, *J* = 7.0 Hz, 2H, CH_2), 2.68 (t, *J* = 7.0 Hz, 2H, CH_2), 3.03-3.24 (m, 12H, 1-CH_2, 3-CH_2, 5-CH_2, 7-CH_2, 9-CH_2, 11-CH_2, overlapping); ^{13}C NMR, 125.77 MHz (CD_3OD): 27.5 (9 x CH_3 Boc, 2-CH_2, 6-CH_2, 10-CH_2, overlapping), 29.6 (CH_2), 30.5 (CH_2), 35.4, 36.7,43.2, 44.3 (1-CH_2, 3-CH_2, 5-CH_2, 7-CH_2, 9-CH_2, 11-CH_2, overlapping), 79.2-80.9 (3 x Cq Boc), 155.2-157.4 (3 x C=O Boc), 171.2 (CONH), 173.9 (COOH).

Synthesis of compound 10

A solution of the tri-Boc protected spermine **8** (0.61 g, 1.21 mmol) in anhydrous pyridine (5 mL) under nitrogen was treated with succinic anhydride (0.12 g, 1.21 mmol, 1 equiv.) at 20 °C. The solution was stirred for a further 18 h. The solution was then concentrated *in vacuo*. The crude material was extracted with chloroform (3 x 15 mL). The combined organic extracts were dried (Na_2SO_4), filtered, and concentrated in *vacuo*. The desired product **10** was obtained as a colourless oil (0.63 g, 86%). TLC analysis showed one spot (R_f = 0.4 (EtOAc: ethanol: aq. ammonia (32%); 7:2:1 v/v/v). HRMS: Found 601.3867 (*m/z*), $C_{29}H_{53}N_4O_9$ requires 601.3860 (*m/z*) $[M - H]^-$; IR (film); 2948-3536 (COOH) and 1686 (C=O) cm^{-1}; ^1H NMR, 500 MHz (CD_3OD): 1.41-1.49 (m, 27H, 9 x CH_3 Boc) 1.48-1.53 (m, 4H, 6-CH_2, 7-CH_2), 1.64-1.74 (m, 4H, 2-CH_2, 11-CH_2), 2.45 (t, *J* = 7.0 Hz, 2H, CH_2), 2.58 (t, *J* = 7.0 Hz, 2H, CH_2), 3.03-3.24 (m, 12H, 1-CH_2, 3-CH_2, 5-CH_2, 8-CH_2, 10-CH_2, 12-CH_2, overlapping); ^{13}C NMR, 125.77 MHz (CD_3OD): 25.5, 26.8 (6-CH_2, 7-CH_2), 27.5 (9 x CH_3 Boc), 26.9 (2-CH_2, 10-CH_2), 28.8 (CH_2), 30.2 (CH_2), 36.3, 36.7, 39.9, 44.1 (1-CH_2, 3-CH_2, 5-CH_2, 8-CH_2, 10-CH_2, 12-CH_2, overlapping), 79.5-81.0 (3 x Cq Boc), 156.1-157.5 (3 x C=O Boc), 173.4 (CONH), 174.8 (COOH).

Synthesis of compound 11

A solution of **9** (0.40 g, 0.67 mmol), HBTu (0.25 g, 0.67 mmol, 1 equiv.), and TEA (0.06 g, 0.67 mmol, 1 equiv.) in anhydrous DMF (10 mL) was treated with **7** (0.33 g, 0.67 mmol, 1 equiv.) in anhydrous DMF (3 mL) under nitrogen at 20 °C. The solution was stirred for a further 18 h. The solution was then concentrated in *vacuo*, and the crude material was extracted with chloroform (3 x 15 mL). The combined organic extracts were dried (Na_2SO_4), filtered, concentrated in *vacuo* and purified over silica gel, (DCM: methanol; 9.5:0.5 to 9:1 v/v). After combining fractions and concentrating them, the desired product **11** was obtained as a pale yellow oil (0.47 g, 66%). TLC analysis showed one spot (R_f = 0.5 (DCM: methanol, 9:1 v/v). HRMS: Found 1059.7202 (*m/z*), $C_{52}H_{99}N_8O_{14}$ requires 1059.7224 (*m/z*) $[M + H]^+$; IR (Film); 1693 (C=O) cm^{-1}; ^1H NMR, 500 MHz ($CDCl_3$): 1.40-1.48 (m, 54H, 18 x CH_3 Boc), 1.60-1.79 (m, 12H, 2-CH_2, 6-CH_2, 10-CH_2, overlapping), 2.52 (s, 4H, 14-CH_2), 3.06-3.35 (m, 24H, 1-CH_2, 3-CH_2, 5-CH_2, 7-CH_2, 9-CH_2, 11-CH_2); ^{13}C NMR, 125.77 MHz ($CDCl_3$):

27.4, 28.3 (18 x CH_3 Boc, 2-CH_2, 6-CH_2, 10-CH_2, overlapping), 31.8 (14-CH_2), 35.9, 37.5, 43.7, 44.8 (1-CH_2, 3-CH_2, 5-CH_2, 7-CH_2, 9-CH_2, 11-CH_2), 79.3-79.8 (6 x Cq Boc), 155.3-157.7 (6 x C=O Boc), 172.6 (2 x NHCO).

Synthesis of compound 12

A solution of **10** (0.35 g, 0.58 mmol), HBTu (0.21 g, 0.58 mmol, 1 equiv.), and TEA (0.05 g, 0.58 mmol, 1 equiv.) in anhydrous DMF (10 mL) was treated with **8** (0.29 g, 0.58 mmol, 1 equiv.) in anhydrous DMF (3 mL) under nitrogen at 20 °C. The solution was stirred for a further 18 h. The solution was then concentrated in *vacuo*, and the crude material was extracted with chloroform (3 x 15 mL). The combined organic extracts were dried (Na_2SO_4), filtered, concentrated in *vacuo* and purified over silica gel, (DCM: methanol; 9.5:0.5 to 9:1 v/v). After combining fractions and concentrating them, the desired product **12** was obtained as a pale yellow oil (0.37 g, 58%). TLC analysis showed one spot (R_f = 0.5 (DCM: methanol, 9: 1 v/v). HRMS: Found 1087.7503 (*m/z*), $C_{54}H_{103}N_8O_{14}$ requires 1087.7516 (*m/z*) $[M + H]^+$; IR (Film); 1673 (C=O) cm^{-1}; ^1H NMR, 500 MHz ($CDCl_3$): 1.40-1.52 (m, 62H, 18 x CH_3 Boc, 6-CH_2, 7-CH_2, overlapping), 1.61-1.71 (m, 8H, 2-CH_2, 11-CH_2, overlapping), 2.52 (s, 4H, 15-CH_2), 3.10-3.30 (m, 24H, 1-CH_2, 3-CH_2, 5-CH_2, 8-CH_2, 10-CH_2, 12-CH_2, overlapping); ^{13}C NMR, 125.77 MHz ($CDCl_3$): 25.7 (6-CH_2, 7-CH_2), 28.8 (18 x CH_3 Boc, 2-CH_2, 11-CH_2, overlapping), 31.6 (15-CH_2), 36.4, 37.9, 43.4, 46.6 (1-CH_2, 3-CH_2, 5-CH_2, 8-CH_2, 10-CH_2, 12-CH_2, overlapping), 79.7 (6 x Cq Boc), 155.7-156.9 (6 x C=O Boc), 173.1 (2 x NHCO).

Synthesis of compound 13

A solution of **9** (0.30 g, 0.49 mmol), HBTu (0.18 g, 0.49 mmol, 1 equiv.), and TEA (0.04 g, 0.49 mmol, 1 equiv.) in anhydrous DMF (10 mL) was treated with **8** (0.24 g, 0.49 mmol, 1 equiv.) in anhydrous DMF (3 mL) under nitrogen at 20 °C. The solution was stirred for a further 18 h. The solution was then concentrated in *vacuo*, and the crude material was extracted with chloroform (3 x 15 mL). The combined organic extracts were dried (Na_2SO_4), filtered, concentrated in *vacuo* and purified over silica gel, (DCM: methanol; 9.5:0.5 to 9:1 v/v). After combining fractions and concentrating them, the desired product **13** was obtained as a pale yellow oil (0.22 g, 41%). TLC analysis showed

one spot (R_f = 0.5 (DCM: methanol, 9:1 v/v). HRMS: Found 1073.7359 (*m/z*), $C_{53}H_{101}N_8O_{14}$ requires 1073.7352 (*m/z*) $[M + H]^+$; IR (Film); 1693 (C=O) cm^{-1}; ^1H NMR, 500 MHz ($CDCl_3$): 1.39-1.54 (m, 58H, 18x CH_3 Boc, 23-CH_2, 24-CH_2, overlapping), 1.58-1.78 (m, 10H, 2-CH_2, 6-CH_2, 10-CH_2, 19-CH_2, 28-CH_2, overlapping), 2.53 (s, 4H, 14-CH_2, 15-CH_2), 3.06-3.33 (m, 24H, 1-CH_2, 3-CH_2, 5-CH_2, 7-CH_2, 9-CH_2, 11-CH_2, 18-CH_2, 20-CH_2, 22-CH_2, 25-CH_2, 27-CH_2, 29-CH_2, overlapping); ^{13}C NMR, 125.77 MHz ($CDCl_3$): 25.6 (23-CH_2, 24-CH_2), 28.4, 27.6, 28.9 (18x CH_3 Boc, 2-CH_2, 6-CH_2, 10-CH_2, 19-CH_2, 28-CH_2, overlapping), 31.9 (14-CH_2, 15-CH_2), 35.9, 37.2, 37.6, 43.4, 44.9, 46.6 (1-CH_2, 3-CH_2, 5-CH_2, 7-CH_2, 9-CH_2, 11-CH_2, 18-CH_2, 20-CH_2, 22-CH_2, 25-CH_2, 27-CH_2, 29-CH_2), 78.8, 79.9 (6 x Cq Boc), 155.0, 156.0 (6 x C=O Boc), 172.36 (2 x NHCO).

Synthesis of compound 14

Compound **11** (0.24 g, 0.22 mmol) was deprotected according to general procedure Boc removal to yield the desired product **14** as a white solid (0.24 g, 99 %). HRMS: Found 459.4058 (*m/z*), $C_{22}H_{51}N_8O_2$ requires 459.4057 (*m/z*) $[M + H]^+$; IR (KBr disc); 1695 (C=O) cm^{-1}; ^1H NMR, 500 MHz (D_2O): 1.84-1.93 (m, 4H, 10-CH_2), 2.05-2.15, (m, 8H, 2-CH_2, 6-CH_2), 2.53 (s, 4H, 2 x CH_2), 3.04-3.21 (m, 20H, 1-CH_2, 3-CH_2, 5-CH_2, 7-CH_2, 9-CH_2, overlapping), 3.27 (t, J = 7.4 Hz, 4H, 11-CH_2); ^{13}C NMR, 125.77 MHz (D_2O): 22.7, 23.7 (2-CH_2, 6-CH_2), 25.5 (10-CH_2), 30.7 (14-CH_2), 36.0 (11-CH_2), 36.4, 44.3, 44.5, 44.6, 45.2 (1-CH_2, 3-CH_2, 5-CH_2, 7-CH_2, 9-CH_2), 116.1 (q, ^1J = 291.3 Hz, CF_3), 162.9 (q, ^2J = 37.6 Hz, *C*O-CF_3), 175.3 (2 x NHCO).

Synthesis of compound 15

Compound **12** (0.18 g, 0.17 mmol) was deprotected according to general procedure Boc removal to yield the desired product **15** as a white solid (0.18 g, 99 %). HRMS: Found 487.4372 (*m/z*), $C_{24}H_{55}N_8O_2$ requires 487.4370 (*m/z*) $[M + H]^+$; IR (KBr disc); 1688 (C=O) cm^{-1}; ^1H NMR, 500 MHz (D_2O): 1.75-1.80 (m, 8H, 6-CH_2, 7-CH_2), 1.86-1.92 (m, 4H, 11-CH_2), 2.04-2.13 (m, 4H, 2-CH_2), 2.53 (s, 4H,15-CH_2), 3.02-3.20 (m, 20H, 1-CH_2, 3-CH_2, 5-CH_2, 8-CH_2, 10-CH_2), 3.26 (t, J = 7.0 Hz, 4H, 12-CH_2); ^{13}C NMR, 125.77 MHz (D_2O): 22.7 (6-CH_2, 7-CH_2), 23.7 (2-CH_2), 25.5 (11-CH_2), 30.7 (15-

CH_2), 36.0 (12-CH_2), 36.4, 44.4, 45.1, 46.7, 46.9 (1-CH_2, 3-CH_2, 5-CH_2, 8-CH_2, 10-CH_2), 116.3 (q, ^1J = 292.0 Hz, CF_3), 161.7 (q, ^2J = 36.6 Hz, *C*O-CF_3), 175.3 (2 x NHCO).

Synthesis of compound 16

Compound **13** (0.22 g, 0.20 mmol) was deprotected according to general procedure Boc removal to yield the desired product **16** as a white solid (0.22 g, 99 %). HRMS: Found 473.4213 (*m/z*), $C_{23}H_{53}N_8O_2$ requires 473.4212 (*m/z*) $[M + H]^+$; IR (KBr disc); 1683 (C=O) cm^{-1}; ^1H NMR, 500 MHz (D_2O): 1.71-1.78 (m, 4H, 23-CH_2, 24-CH_2), 1.83-1.91 (m, 4H, 10-CH_2, 19-CH_2), 2.02-2.14 (m, 6H, 2-CH_2, 6-CH_2, 28-CH_2), 2.53 (s, 4H, 14-CH_2, 15-CH_2), 2.85-3.13 (m, 20H, 1-CH_2, 3-CH_2, 5-CH_2, 7-CH_2, 9-CH_2, 20-CH_2, 22-CH_2, 25-CH_2, 27-CH_2, 29-CH_2), 3.27 (t, *J* = 7.4 Hz, 4H, 11-CH_2, 18-CH_2); ^{13}C NMR, 125.77 MHz (D_2O): 22.6, 22.7 (23-CH_2, 24-CH_2), 23.5 (2-CH_2, 6-CH_2, 28-CH_2), 25.5 (10-CH_2, 19-CH_2), 30.7 (14-CH_2, 15-CH_2), 36.0 (11-CH_2, 18-CH_2), 36.4, 44.3, 44.4, 44.5, 44.6, 45.0, 45.2, 46.8, 46.9 (1-CH_2, 3-CH_2, 5-CH_2, 7-CH_2, 9-CH_2, 20-CH_2, 22-CH_2, 25-CH_2, 27-CH_2, 29-CH_2), 116.5 (q, ^1J = 291.0 Hz, CF_3), 163.1 (q, ^2J = 38.0 Hz, *C*O-CF_3), 175.3 (2 x NHCO).

Synthesis of compound 18

To a solution of norspermidine **17** (0.50 g, 3.81 mmol) in ethanol (10 mL) was treated with acrylonitrile (0.60 g, 11.4 mmol, 3 equiv.) at 25 °C. The solution was stirred for a further 48 h. The solution was then concentrated in *vacuo*, and the crude material was purified over silica gel, (DCM: methanol; 9.9:0.1 to 9:1 v/v). After combining fractions and concentrating them, the desired product **18** was obtained as a yellow oil (0.331 g, 30%). TLC analysis showed one spot (R_f = 0.4 (DCM: methanol, 9:1 v/v). HRMS: Found 291.2342 (*m/z*), $C_{15}H_{27}N_6$ requires 291.2292 (*m/z*) $[M + H]^+$; IR (Film); 2250 (CN) cm^{-1}; ^1H NMR, 500 MHz ($CDCl_3$): 1.62-1.69 (m, 4H, 6-CH_2), 2.47-2.57 (m, 10H, 2 x 2-CH_2, 2 x 7-CH_2, 10-CH_2), 2.68-2.76 (m, 6H, 2 x 5-CH_2, 9-CH_2), 2.92 (t, *J* = 6.5 Hz, 4H, 2 x 3-CH_2); ^{13}C NMR, 125.77 MHz ($CDCl_3$): 16.7 (10-CH_2), 18.7 (2 x 2-CH_2), 27.4 (2 x 6-CH_2), 45.1 (2 x 3-CH_2), 47.2 (2 x 5-CH_2), 49.5 (9-CH_2), 51.7 (2x 7-CH_2), 118.9 (1-CN), 119.2 (2 x 11-CN).

Synthesis of compound 19

To a solution of norspermidine **17** (0.50 g, 3.81 mmol) in ethanol (10 mL) was treated with acrylonitrile (1.01 g, 19.0 mmol, 5 equiv.) at 20°C. The solution was stirred for a further 72 h. The solution was then concentrated in *vacuo*, and the crude material was purified over silica gel, (DCM: methanol; 10:0 to 9:1 v/v). After combining fractions and concentrating them, the desired product **19** was obtained as a yellow oil (0.60 g, 40%). TLC analysis showed one spot (R_f = 0.6 (DCM: methanol, 9:1 v/v). HRMS: Found 397.2761 (*m/z*), $C_{21}H_{33}N_8$ requires 397.2750 (*m/z*) $[M + H]^+$; IR (Film) 2254 (CN) cm^{-1}; ^1H NMR, 500 MHz ($CDCl_3$): 1.60-1.70 (m, 4H, 2x CH_2), 2.51 (t, J = 6.9 Hz, 10H, 4 x 2-CH_2, 10-CH_2), 2.56 (t, J = 6.9 Hz, 4H, 2 x 7-CH_2), 2.63 (t, J = 6.9 Hz, 4H, 2 x 5-CH_2), 2.74 (t, J = 6.9 Hz, 2H, 9-CH_2), 2.86 (t, J = 6.9 Hz, 8H, 4 x 3-CH_2); ^{13}C NMR, 125.77 MHz ($CDCl_3$): 16.8 (10-CH_2), 16.9 (4x 2-CH_2), 25.3 (2x 6-CH_2), 49.2 (9-CH_2), 49.5 (4x 3-CH_2), 51.1 (2 x 5-CH_2), 51.2 (2x 7-CH_2), 118.8 (4x 1-CN), 119.5 (11-CN).

Synthesis of compound 22

To a solution of **18** (0.33 g, 1.14 mmol) and NaOH (0.13 g, 3.42 mmol, 3 equiv.) in ethanol (15 mL). Raney nickel (~ 0.5 g) was added to the mixture. The atmosphere over the solution was evacuated and replaced with N_2 gas three times, and then replaced with H_2. The solution was stirred under H_2 for a further 18 h at 20°C. The solution mixture was then filtered through Celite with ethanol. Without further purification, $(Boc)_2O$ (2.48 g, 11.4 mmol, 10 equiv.) was added to the ethanolic solution. The solution was stirred for a further 2 h at 20°C. The solution was then concentrated in *vacuo*, and the crude material was extracted with chloroform (5 x 15 mL) in order to remove the NaOH. The combined organic extracts were dried (Na_2SO_4), filtered, and concentrated in *vacuo*. The desired product **20** was obtained as a pale yellow oil. After combining fractions and concentrating them, the desired product **20**, without further analysis, was deprotected according to general procedure Boc removal to yield the desired product **22** as a pale yellow oil (0.44 g, 40 %). HRMS: Found 303.3158 (*m/z*), $C_{15}H_{39}N_6$ requires 303.3098 (*m/z*) $[M + H]^+$; ^1H NMR, 500 MHz (D_2O): 1.76-1.96 (m, 10H, 2 x 2-CH_2, 2 x 6-CH_2, 10-CH_2), 2.81 (t, J = 8.0 Hz, 6H, 2 x 7-CH_2, 9-CH_2), 2.85-2.94 (m, 8H, 2 x 3-CH_2, 2 x 5-CH_2), 3.02-3.10 (m, 6H, 2 x 1-CH_2, 11-CH_2); ^{13}C NMR, 125.77 MHz (D_2O): 20.2, 23.5 (2 x 2-

CH_2, 2 x 6-CH_2, 10-CH_2), 35.9, 36.1 (2 x 7-CH_2, 9-CH_2), 44.1, 44.5 (2 x 3-CH_2, 2 x 5-CH_2), 49.5 (2 x 1-CH_2, 11-CH_2), 115.0 (q, ^1J = 293.0 Hz, CF_3), 162.2 (q, ^2J = 36.4 Hz, *C*O-CF_3).

Synthesis of compound 23

To a solution of **19** (0.60 g, 1.51 mmol) and NaOH (0.18 g, 4.54 mmol, 3 equiv.) in ethanol (15 mL) was added Raney nickel (~ 0.5 g). The atmosphere over the solution was evacuated and replaced with N_2 gas three times, and then replaced with H_2. The solution was stirred under H_2 for 18 h at 20°C. The mixture was then filtered through Celite with ethanol. Without further purification, $(Boc)_2O$ (3.29 g, 15.1 mmol, 10 equiv.) was added to the ethanolic solution. The solution was stirred for a further 2 h at 20 °C. The solution was then concentrated in *vacuo*, and the crude material was extracted with chloroform (5 x 15 mL) in order to remove the NaOH. The combined organic extracts were dried (Na_2SO_4), filtered, and concentrated in *vacuo*. The desired product **21** was obtained as a pale yellow oil. After combining fractions and concentrating them, the desired product **21**, without further analysis, was deprotected according to general procedure Boc removal to yield the desired product **23** as a pale yellow oil (1.10 g, 55 %). HRMS: Found 417.4357 (*m/z*), $C_{21}H_{53}N_8$ requires 417.4315 (*m/z*) $[M + H]^+$; ^1H NMR, 500 MHz (D_2O): 2.12-2.21 (m, 10H, 4 x 2-CH_2, 10-CH_2), 2.24-2.32 (m, 4H, 2 x 6-CH_2), 3.07-3.15 (m, 10H, 4 x 1-CH_2, 11-CH_2), 3.30-3.42 (m, 18H, 4 x 3-CH_2, 2 x 7-CH_2, 2 x 5-CH_2, 9-CH_2); ^{13}C NMR, 125.77 MHz (D_2O): 18.7 (2 x 6-CH_2), 21.4 (4 x 2-CH_2, 10-CH_2), 36.2 (4 x 1-CH_2, 11-CH_2), 49.9 (4 x 3-CH_2, 2 x 7-CH_2, 2 x 5-CH_2, 9-CH_2), 116.1 (q, ^1J = 290.5 Hz, CF_3), 163.0 (q, ^2J = 38.1 Hz, *C*O-CF_3).

Supplementary Figures

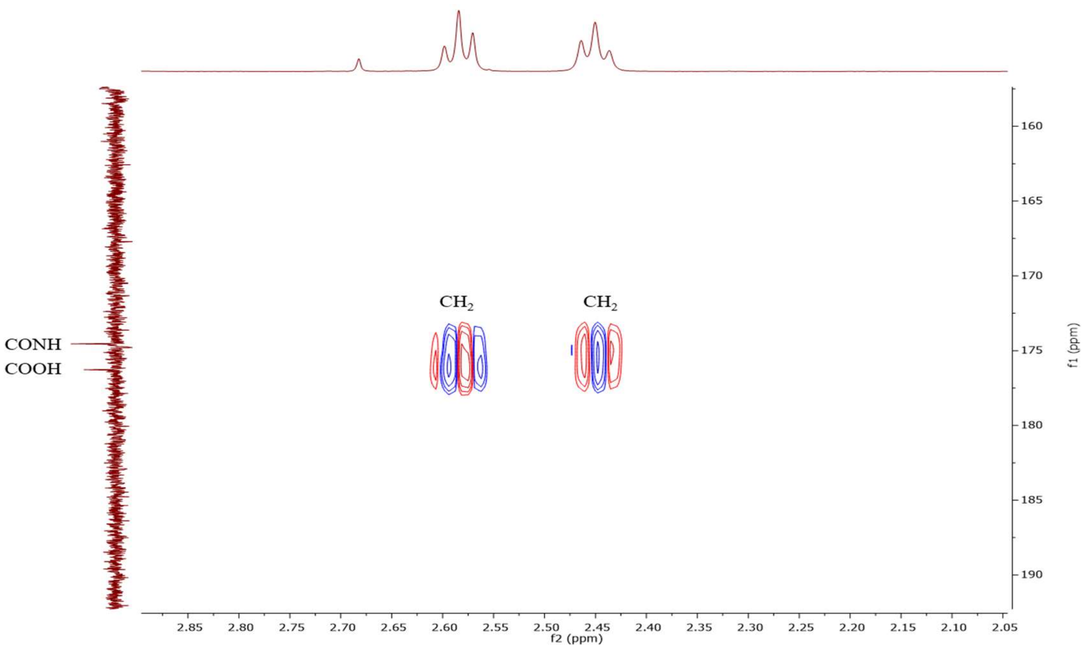

Suppl Figure 1. ^{1}H-^{13}C HMBC NMR spectrum of compound **10** referenced to TMS in 99.8% CD_3OD at 25 °C.

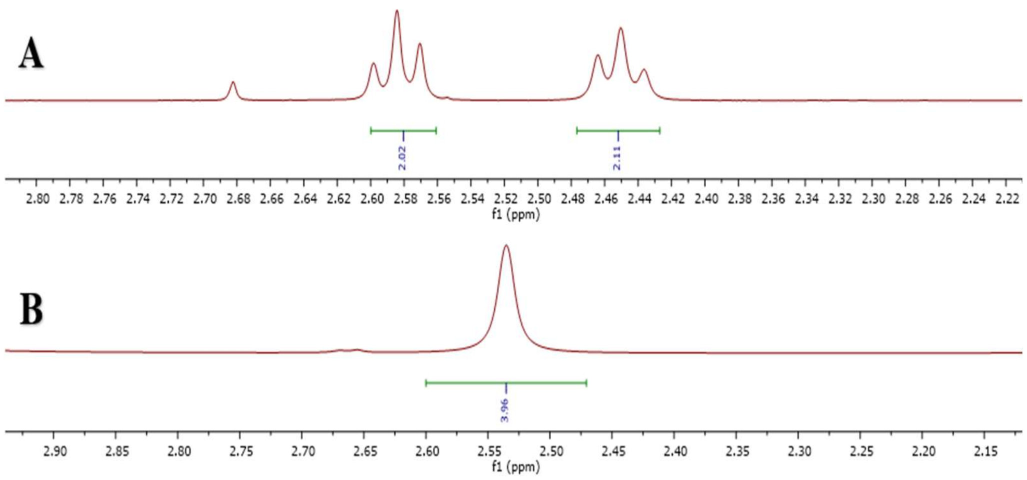

Suppl Figure 2. The ^{1}H NMR spectra of **A 10** and **B 12** referenced to TMS in 99.8% CD_3OD and $CDCl_3$, respectively, at 25 °C.

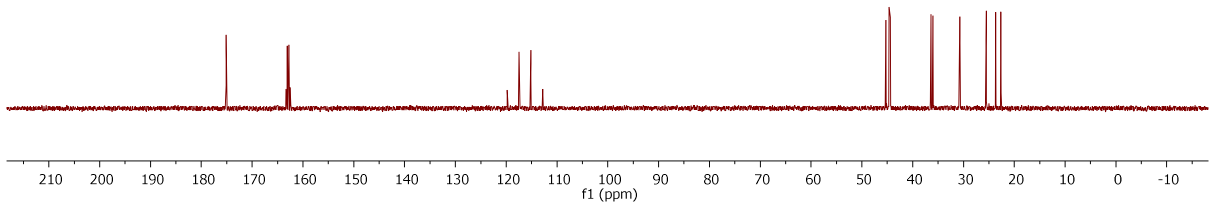

The ^{13}C NMR spectrum of compound **14**

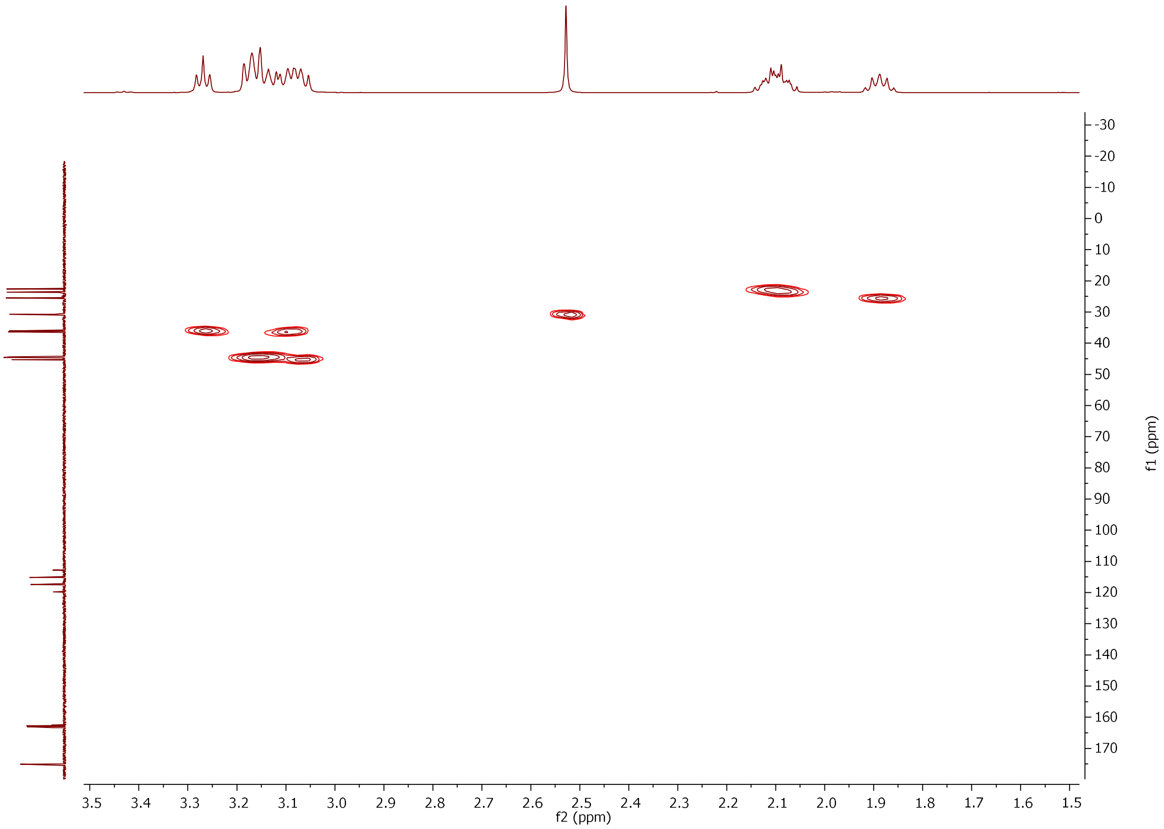

The ^{1}H-^{13}C HSQC NMR spectrum of compound **14**

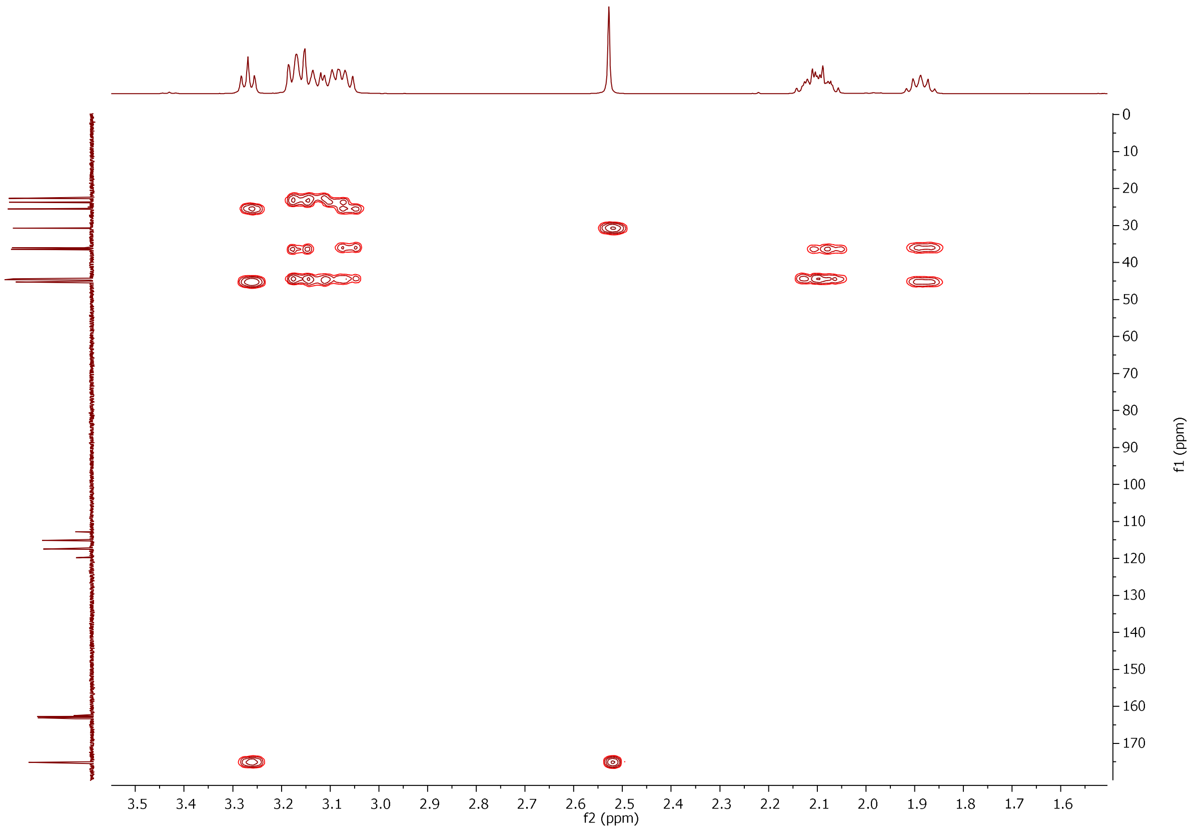

The ^{1}H-^{13}C HMBC NMR spectrum of compound **14**

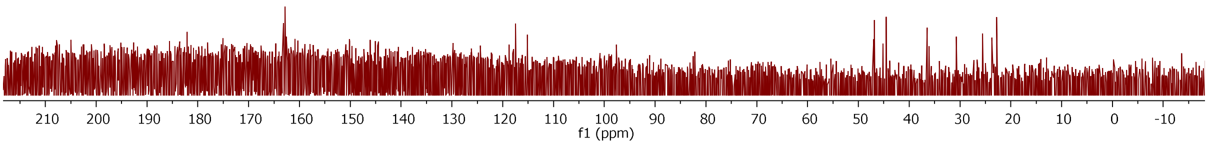

The ^{13}C NMR spectrum of compound **15**

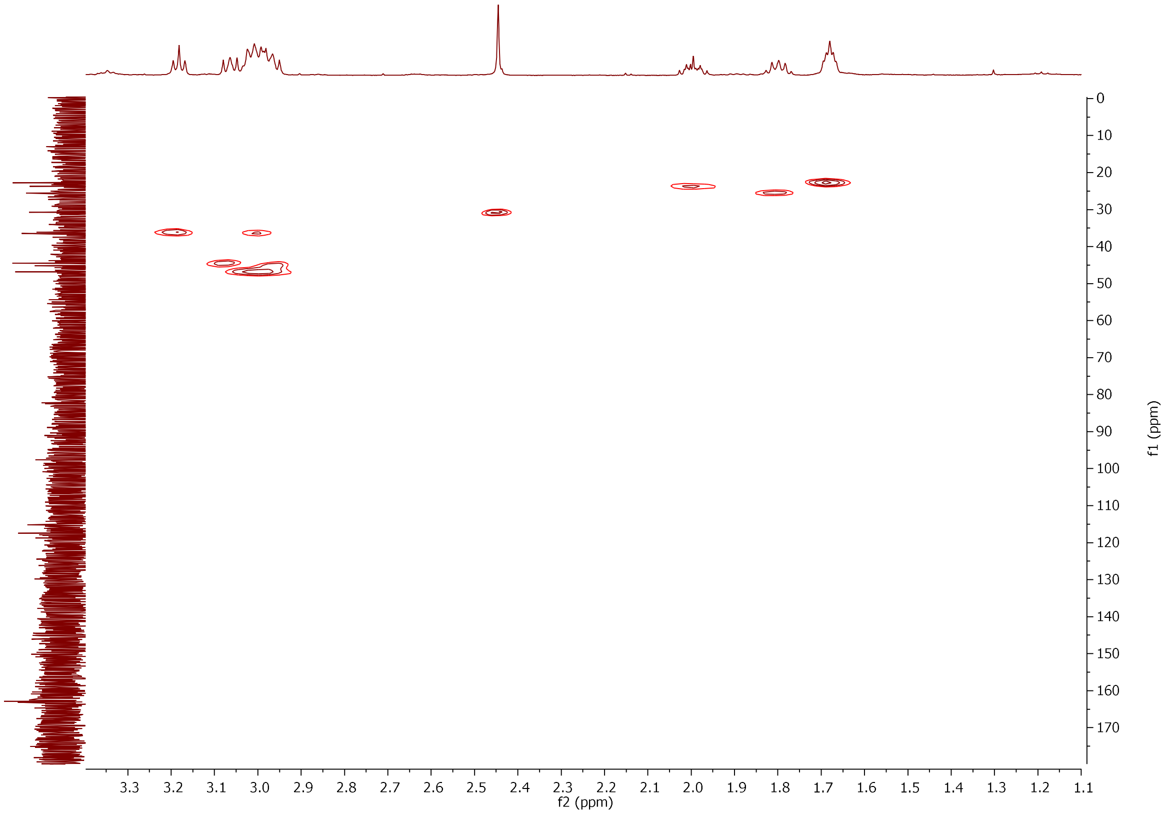

The ^{1}H-^{13}C HSQC NMR spectrum of compound **15**

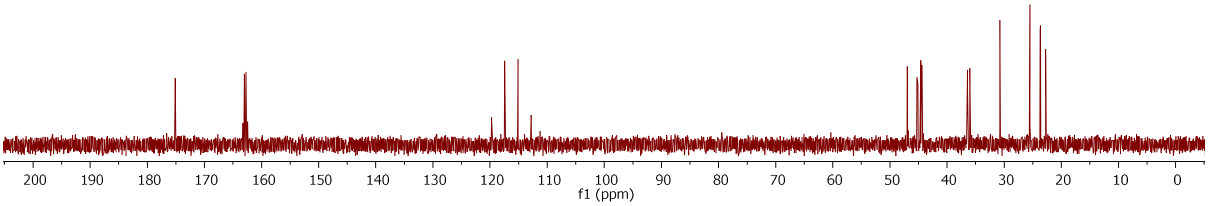

The ^{13}C NMR spectrum of compound **16**

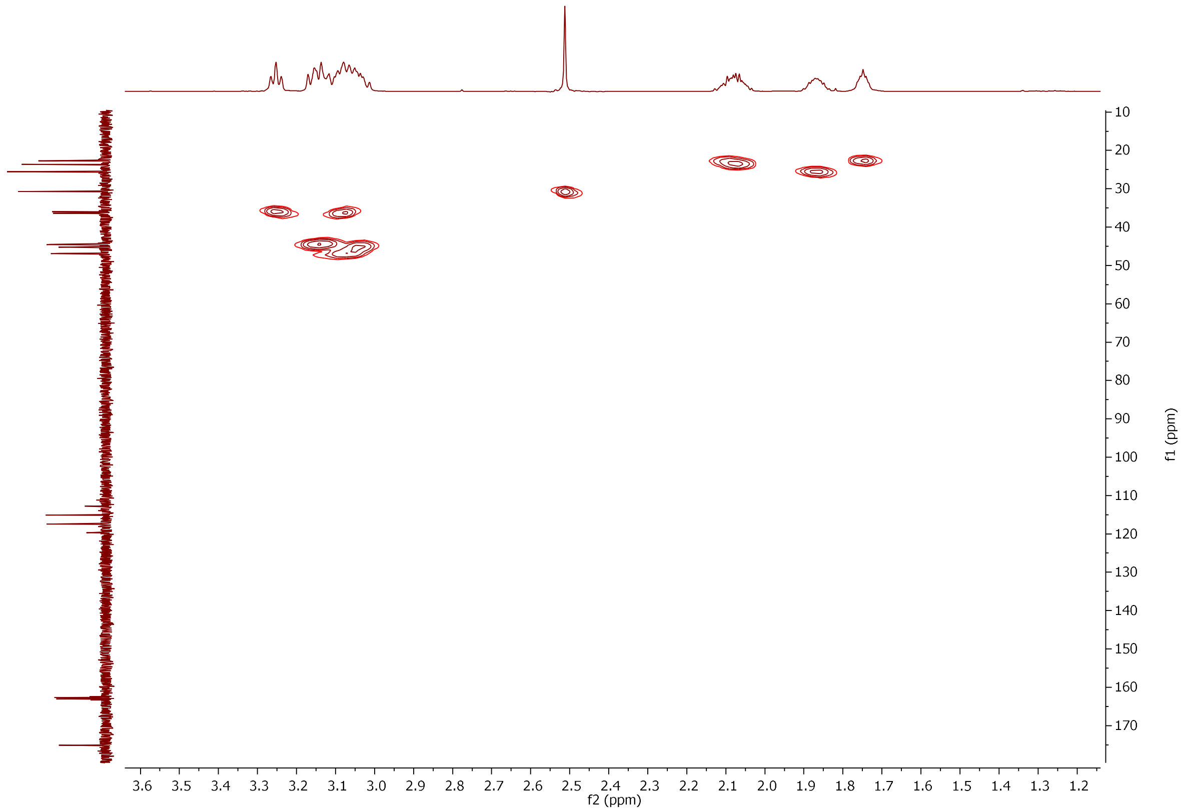

The ^{1}H-^{13}C HSQC NMR spectrum of compound **16**

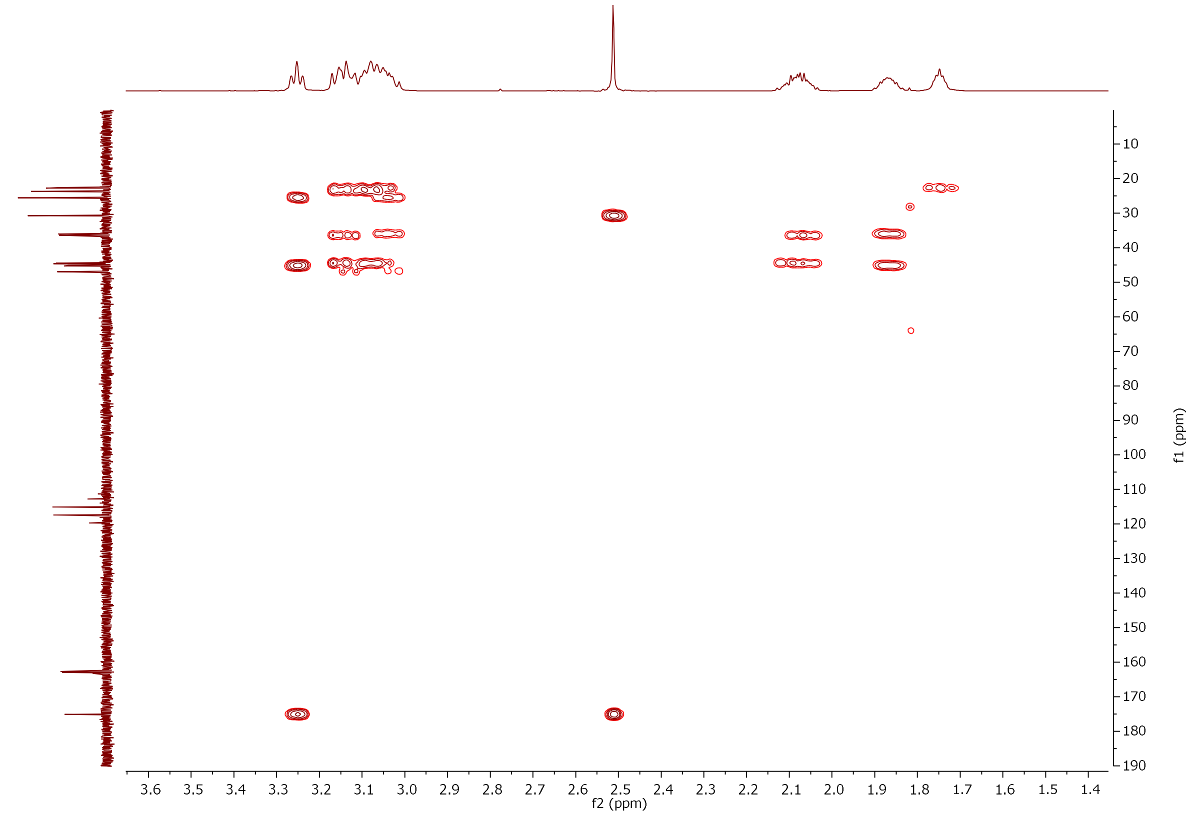

The ^{1}H-^{13}C HMBC NMR spectrum of compound **16**

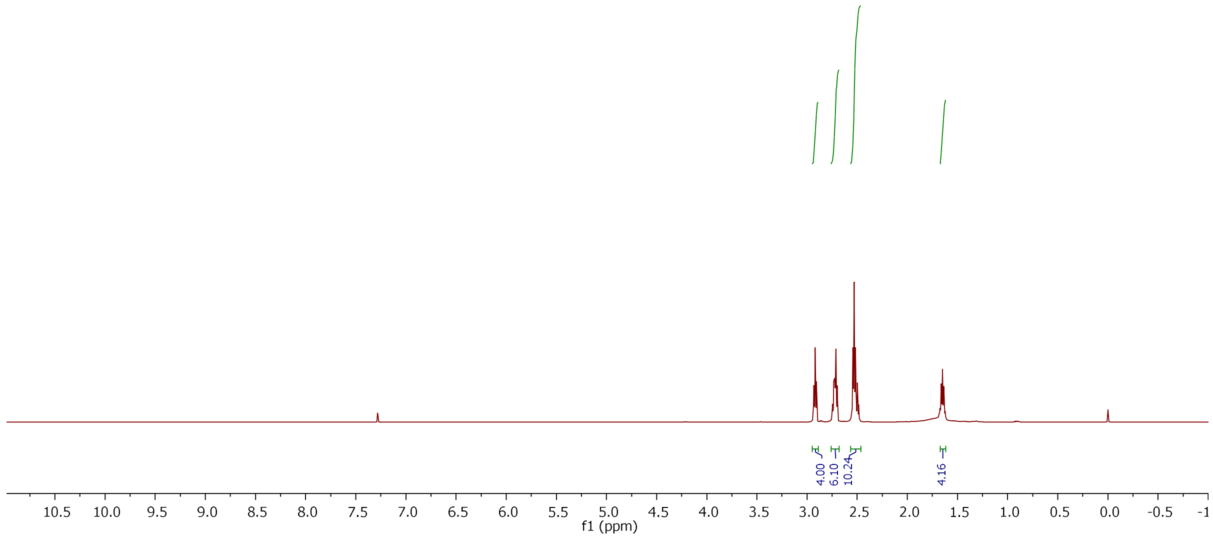

The ^{1}H NMR spectrum of compound **18**

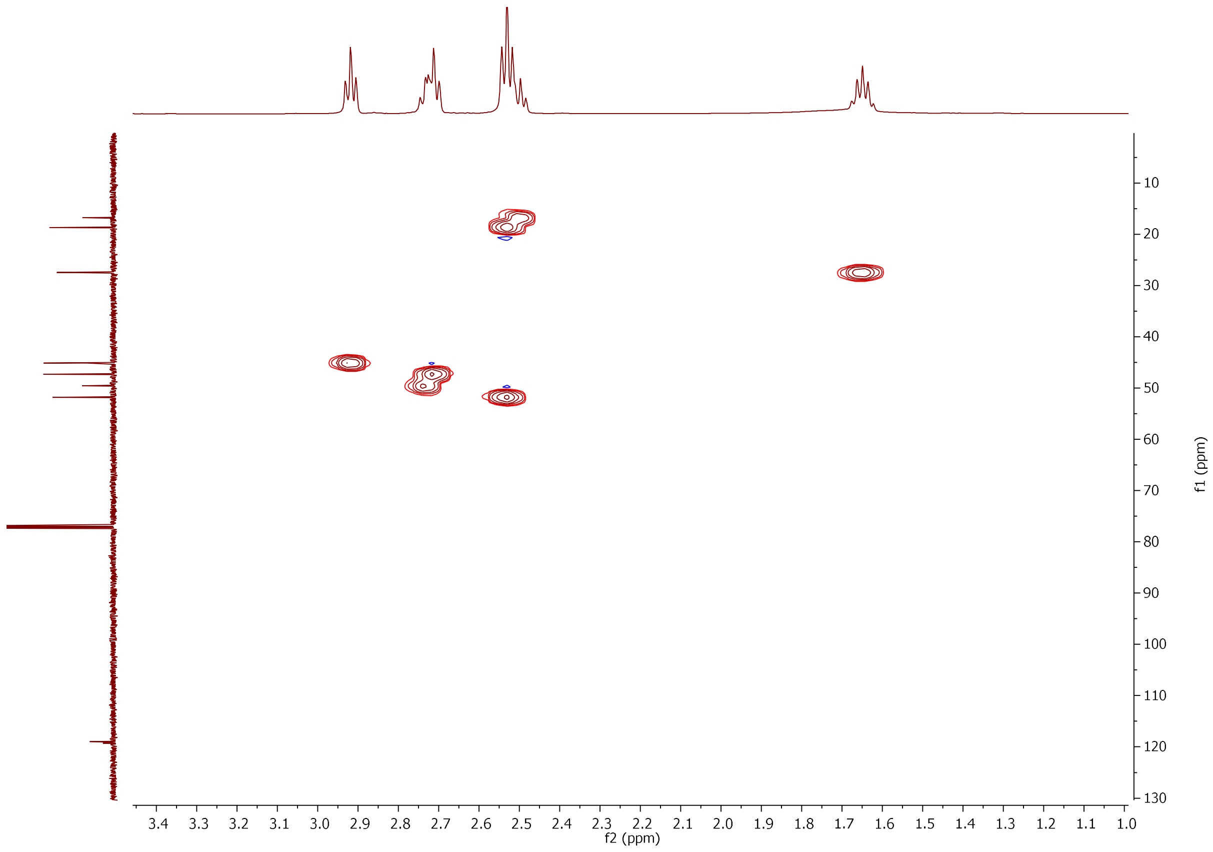

The ^{1}H-^{13}C HSQC NMR spectrum of compound **18**

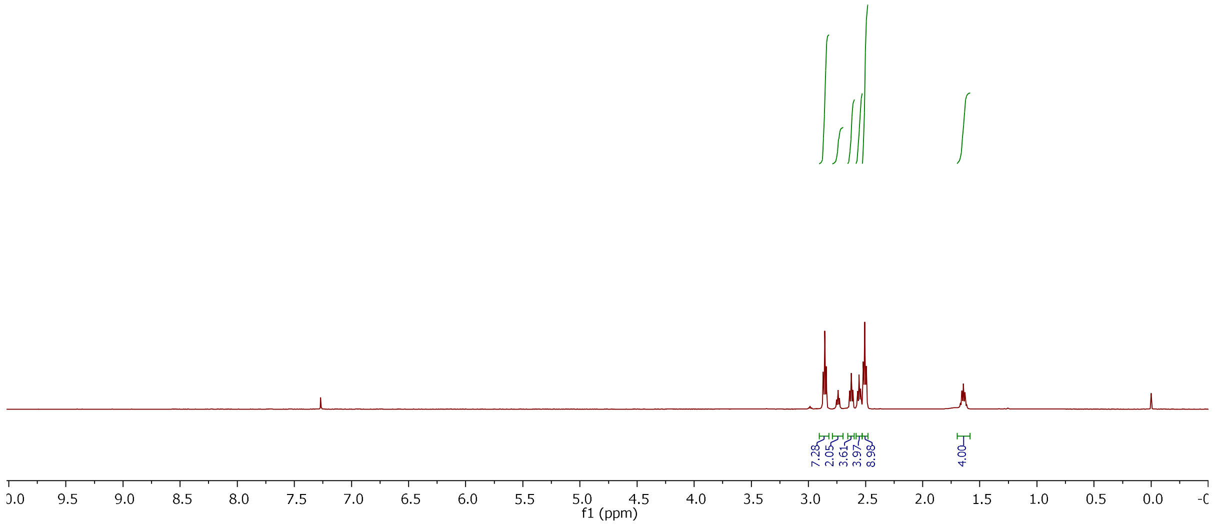

The ^{1}H NMR spectrum of compound **19**

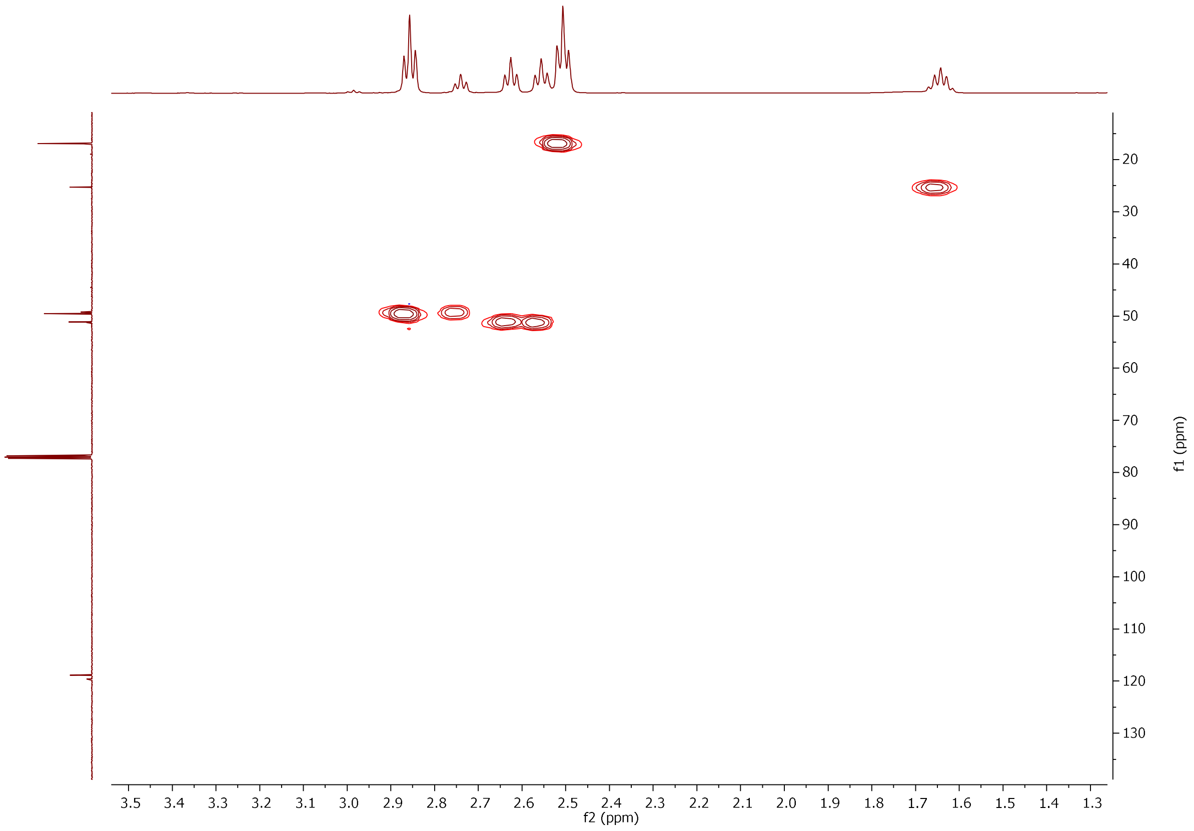

The ^{1}H-^{13}C HSQC NMR spectrum of compound **19**

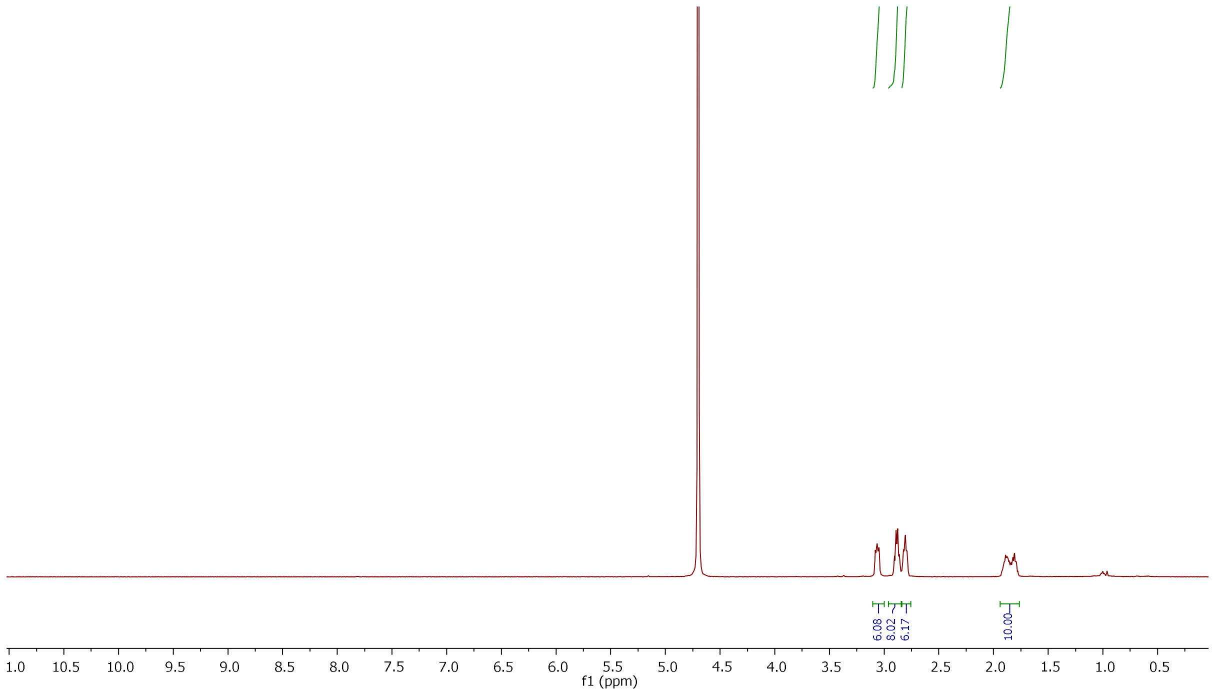

The ^{1}H NMR spectrum of compound **22**

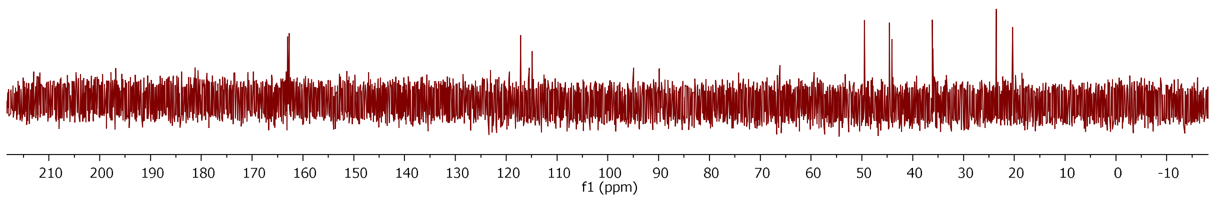

The ^{13}C NMR spectrum of compound **22**

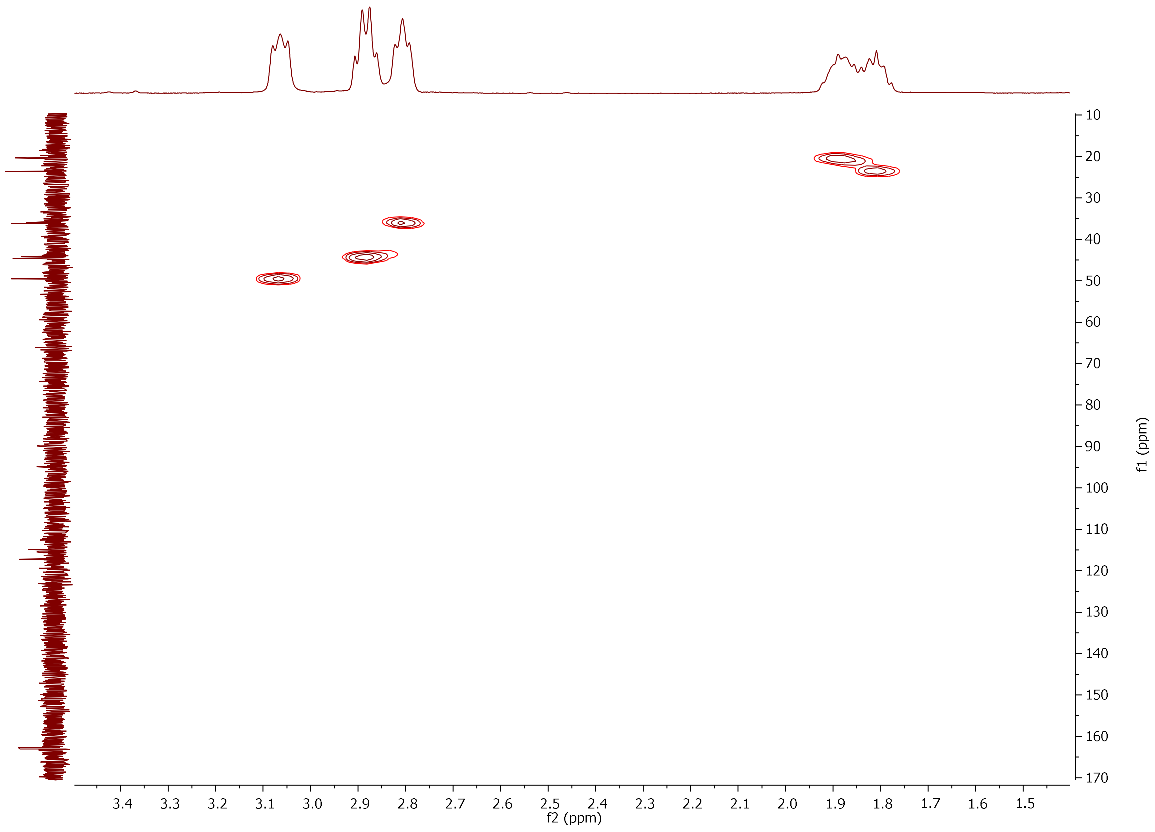

The ^{1}H-^{13}C HSQC NMR spectrum of compound **22**

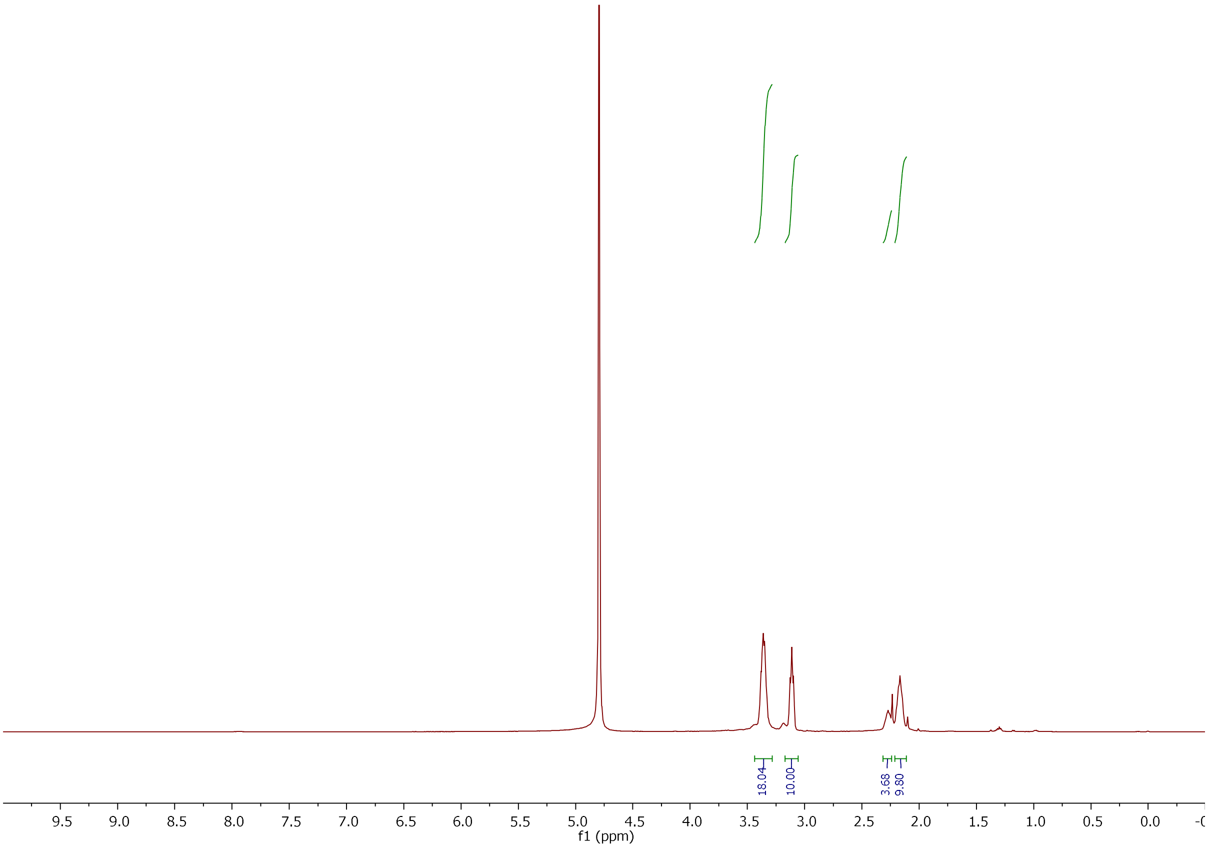

The ^{1}H NMR spectrum of compound **23**

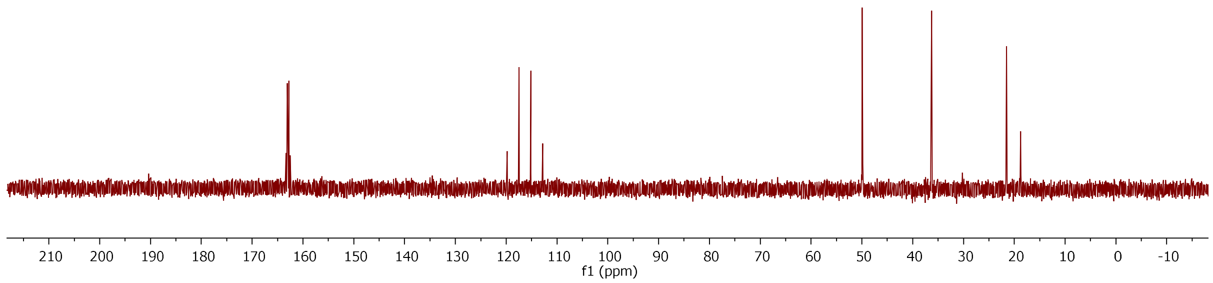

The ^{13}C NMR spectrum of compound **23**

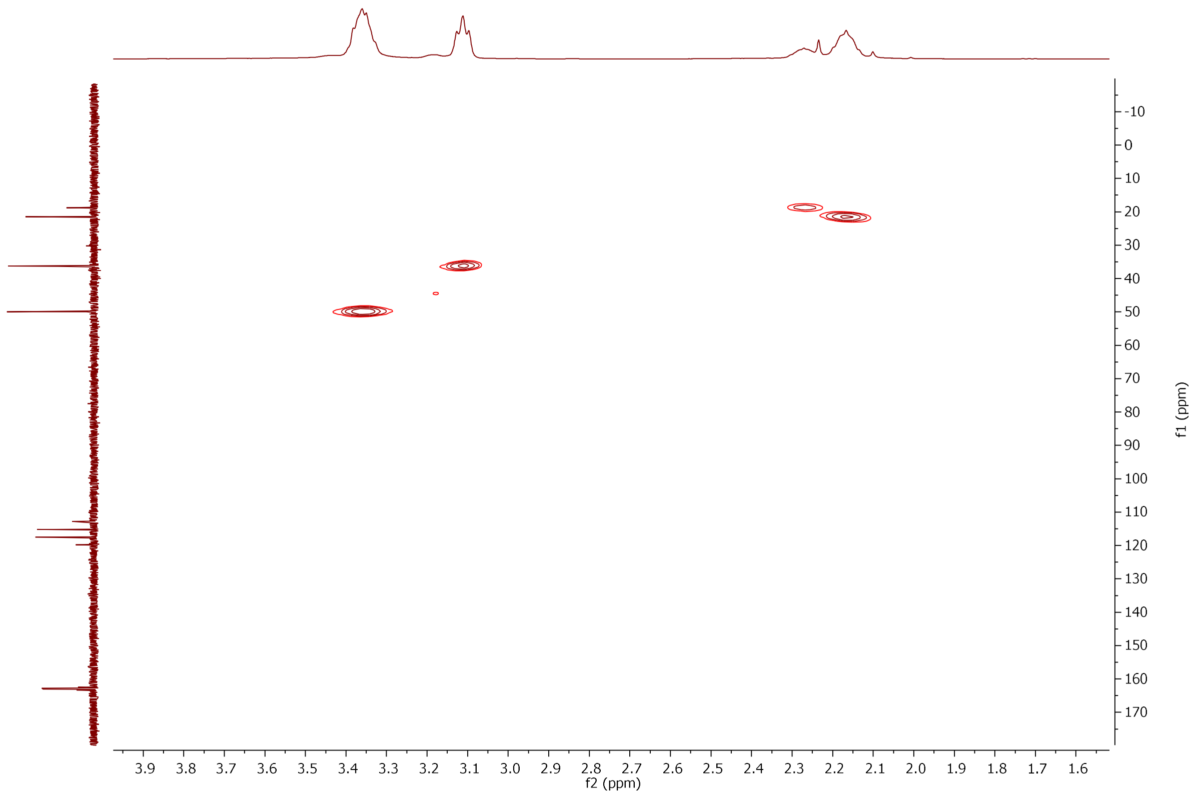

The ^{1}H-^{13}C HSQC NMR spectrum of compound **23**
